# Structure and Spectroscopy of Free Base, Copper, and Zinc Tetrapentylporphyrin

**DOI:** 10.1021/acs.jpca.6c02509

**Published:** 2026-06-09

**Authors:** Breanna E. Muldowney, Nneka Damola Ajayi, Wei-Yuan Chen, G. Richard Geier, Christopher J. Ziegler, Victor N. Nemykin

**Affiliations:** † Department of Chemistry, 8664 University of Tennessee, Knoxville, Tennessee 37996, United States; ‡ Department of Chemistry, 1076 University of Akron, Akron, Ohio 44325, United States; § Department of Chemistry, 3719 Colgate University, Hamilton, New York 13346, United States

## Abstract

Synthetic porphyrins have been crucial as models for heme units and other biological porphyrins, as well as components of advanced materials and catalysts. Much of the work on synthetic porphyrins has focused on the meso-substituted 5,10,15,20-tetraphenylporphyrin (TPP) or the pyrrole-substituted 2,3,7,8,12,13,17,18-octaethylporphyrin (OEP). In this report, we present a comprehensive spectroscopic, electrochemical, and computational study of free-base, zinc­(II), and copper­(II) 5,10,15,20-tetrapentylporphyrin (TPeP). TPeP can be prepared via a two-step, one-flask reaction of pyrrole and hexanal mediated by Montmorillonite K10, followed by oxidation with 2,3-dichloro-5,6-dicyano-1,4-benzoquinone (DDQ) in yields of around 40%. Similar to TPP, the TPeP systems have the highest occupied molecular orbital (HOMO) with *a*_*2u*_ symmetry and are, in general, easier to oxidize than their corresponding TPP or OEP analogues.

## Graphical abstract

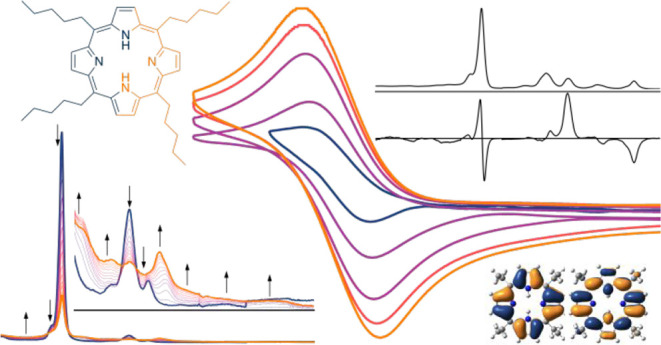

## Introduction

For most cellular organisms, hemes and related pigments are essential to life.^1–3^ The heme cofactor contains a porphyrin macrocycle and a metal-chelating heterocycle with an 18-electron annulene ring. Along with the cofactors found in heme proteins, related tetrapyrrolic macrocycles are found across biology, ranging from the chlorophyll family of light-harvesting chromophores^4,5^ to the B12 prosthetic groups essential for biosynthesis.^6,7^ Our understanding of many of the fundamental physical and chemical properties of biological porphyrinoids has come from the study of simple synthetic porphyrins as models.^8–13^ Synthetic porphyrins, due to their readily available syntheses, have greatly advanced our knowledge of biological systems and have allowed for the extension of this chemistry to numerous nonbiological applications.^14–19^

The field of synthetic porphyrins began in the 1930s with the discovery of 5,10,15,20-tetraphenylporphyrin (TPP) by Rothemund (Figure 1).^20–23^ Over the subsequent decades, improved methods of synthesis significantly expanded investigations into TPP.^24–26^ Unsubstituted free-base H_2_TPP can be produced in one step from inexpensive reagents by use of the Adler condensation, or alternatively, higher yields and gentler conditions are available via Lindsey conditions.^27,28^ The TPP macrocycle can be structurally altered at the periphery either through modification of reaction conditions or via subsequent synthetic modification. The ready use of TPP and its derivatives has led to major advancements in our understanding of heme chemistry in biology.^29–33^ Additionally, TPP’s ease of synthesis, synthetic utility across the periodic table, and extreme stability have resulted in multiple applications outside of biology, ranging from light and energy harvesting applications^34–36^ to many examples as catalysts for organic transformations.^37–39^

**Figure 1:**
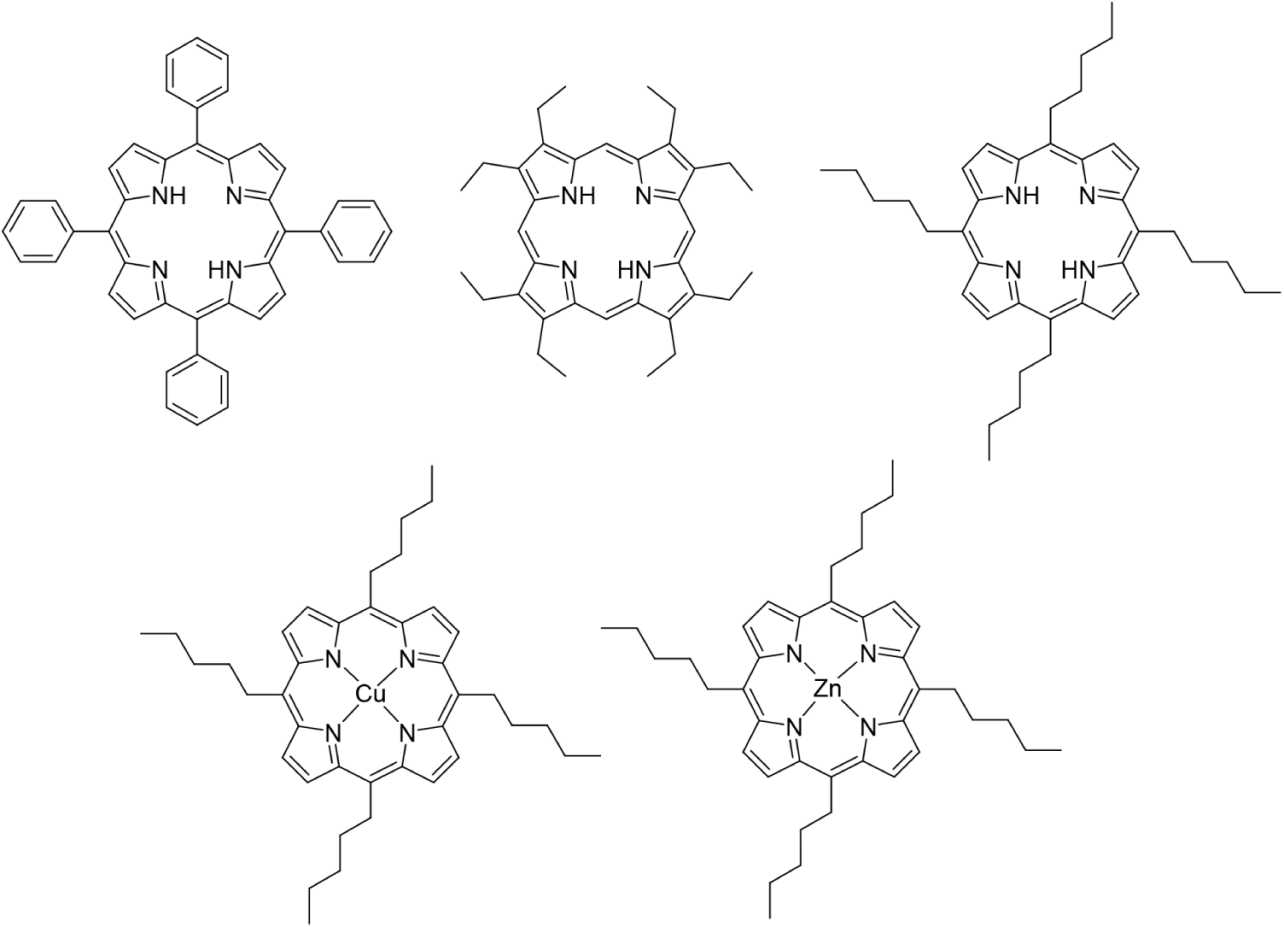
Top: Structures of H_2_TPP (left), H_2_OEP (center), and H_2_TPeP (right). Bottom: The Cu­(II) and Zn­(II) complexes of TPeP.

In 1968, an alternative synthetic porphyrin, 2,3,7,8,12,13,17,18-octaethylporphyrin (OEP), was introduced, which has eight ethyl groups substituted at the pyrrolic β positions (Figure 1).^40^ Advancements in the chemistry of precursor pyrrole compounds, specifically α-substituted 3,4 diethylpyrroles, obviated the need for aldehydes in the macrocycle condensation reaction.^41,42^ Similar routes developed by Barton and Zard can be used to produce a variety of octa-substituted porphyrins at the pyrrole 3,4 positions.^43,44^ The development of OEP provided an important structural contrast to that of TPP; the steric bulk of the phenyl rings was eliminated, and the substitution pattern more closely matches that of biological porphyrins.^45–47^ OEP and similarly alkyl β-substituted porphyrins also exhibit different optical and electronic properties from TPP, with blue-shifted transitions and more negative oxidation and reduction potentials.^48^

In this paper, we present a comprehensive study of a meso-alkylated synthetic porphyrin, 5,10,15,20 tetrapentylporphyrin (TPeP) (Figure 1). In contrast to TPP and OEP, fewer researchers have probed the fundamental properties of meso-alkylated porphyrins. We decided to examine the electronic structure of the H_2_TPeP free base and two metal adducts (Cu­(II) and Zn­(II)) using spectroscopy (UV–visible and magnetic circular dichroism), electrochemistry, spectroelectrochemistry, and computational methods. As seen with TPP and OEP, the location and identity of functional groups induce subtle but readily observable differences in porphyrinic electronic structure. In general, the properties of free-base and metalated TPeP are more similar to the meso-substituted TPP systems. In particular, we observe data consistent with a Gouterman four-orbital model with a HOMO that has *a*_*2u*_ symmetry and a HOMO–1 with *a*_*1u*_ symmetry.^49–52^ Additionally, the TPeP systems are, in general, easier to oxidize than their corresponding TPP or OEP analogues.

## Experimental Section

### General Experimental Methods

^1^H NMR (400 MHz) was collected routinely. Column chromatography was performed on silica (Sorbent Technologies, standard grade, 230–400 mesh, 60 Å). Pyrrole was distilled from CaH_2_ and stored at −15 °C. The distilled pyrrole was used prior to the appearance of any discoloration. Montmorillonite K10 was activated under vacuum at 120–125 °C for 3 h and stored in a desiccator prior to use. All other chemicals were reagent grade and used as received.

UV–vis-NIR spectroscopy was performed in a 5 mm quartz cell purchased from Firefly Sci using a JASCO-770 spectrometer, and MCD was recorded using a JASCO V-1500 spectropolarimeter with a permanent 1.6 T magnet. Each MCD sample was accumulated three times, each with parallel and antiparallel field. All intensities of MCD data are shown in molar ellipticity per tesla. Electrochemistry was conducted in an argon-purged cell with a CH Instruments potentiostat with a three-electrode setup consisting of a glassy carbon working electrode, a platinum wire auxiliary electrode, and an Ag/AgCl wire pseudoreference electrode. Spectroelectrochemical experiments were conducted in an argon-purged cell using a JASCO V-700 UV–vis-NIR spectrometer and a CH Instruments potentiostat in tandem. Data were collected in a 1 mm quartz cell with a platinum mesh working electrode, a platinum wire auxiliary electrode, and an Ag/AgCl wire pseudoreference electrode.

X-ray intensity data were measured on a Bruker PHOTON II CPAD-based diffractometer with dual Cu/Mo ImuS microfocus optics (Cu Kα radiation, λ = 1.54178 Å, Mo Kα radiation, λ = 0.71073 Å). Crystals were mounted on a cryoloop using Paratone oil and placed under a stream of nitrogen at 100 K (Oxford Cryosystems). The detector was placed at a distance of 5.00 cm from the crystal. The data were corrected for absorption with the SADABS program. The structures were refined using the Bruker SHELXTL Software Package (Version 6.1) and solved using direct methods until the final anisotropic full-matrix least-squares refinement of F^2^ converged. Deposition number 2547164 contains the supplementary crystallographic data for this paper. These data can be obtained free of charge via the joint Cambridge Crystallographic Data Center (CCDC) and Fachinformationszentrum Karlsruhe Access Structures service.

### 5,10,15,20-Tetrapentylporphyrin (H_2_TPeP)

The reaction conditions were performed three times. To an oven-dried 2 L round-bottom flask containing a stir bar were added CH_2_Cl_2_ (800 mL), pyrrole (0.555 mL, 8.00 mmol), and hexanal (0.983 mL, 8.00 mmol). After stirring briefly, the reaction was initiated by the addition of activated montmorillonite K10 acid (16.0 g, 20.0 g/L). The flask was tightly capped, and the reaction mixture was stirred for 2 h at room temperature. At a reaction time of 2 h, the mixture (a brown suspension) was oxidized by the addition of 2,3-dichloro-5,6-dicyano-1,4-benzoquinone (DDQ) (2.00 g, 8.81 mmol) at room temperature. After ∼1 min, triethylamine (20 mL, 0.14 mol,) was added, and the mixture was stirred for 1 h at room temperature. The oxidized reaction mixture was examined by TLC (silica, CH_2_Cl_2_, Rf ∼ 0.6) and filtered through a pad of silica eluted with CH_2_Cl_2_ (100 mL) until H_2_TPeP was no longer detected in the eluent by TLC. The filtrate was concentrated to a sticky, purple/brown film. The impure H_2_TPeP was dissolved with CH_2_Cl_2_ (∼10 mL) and subjected to chromatography (silica, CH_2_Cl_2_). Dark purple fractions containing TPeP were collected and evaporated to dryness (507–537 mg). Crystallization from CH_2_Cl_2_/hexanes with gradual evaporation of the CH_2_Cl_2_ (50–60 °C) gave fine crystals that were stored overnight at −15 °C. [Note: a fine precipitate was instead obtained when the solvent volume was reduced too low. Crystallization became more straightforward once seed crystals were available.] The crystals were collected by vacuum filtration, aided by rinsing with pentane, affording dark purple crystals of H_2_TPeP (464–510 mg, 39–43%).^1^H NMR (CDCl_3_), UV–vis (CH_2_Cl_2_), and LD-MS analyses were consistent with published values.^27^

### (5,10,15,20-Tetrapentylporphyrinato)­zinc­(ii) (Zn­(TPeP))

A 10 mg sample of zinc acetate dihydrate was dissolved in 5 mL methanol and added to a solution of an equivalent mass (10 mg) of freebase H_2_TPeP in chloroform (5 mL). The resultant solution was refluxed for 1 h, and the metalation reaction was monitored by TLC. Upon metalation, the solvent was removed from the reaction solution, and the product was purified by silica column chromatography using 10% ethyl acetate in methylene chloride as the eluent. Yield: 8.9 mg (80%)^1^H NMR (CDCl_3_) and UV–vis spectra were consistent with published values.^53,54^

### (5,10,15,20-Tetrapentylporphyrinato)­copper­(ii) (Cu­(TPeP))

An identical procedure to that used for Zn­(TPeP) was employed, but with copper acetate dihydrate. Yield: 9.2 mg (83%). LD-MS HRMS *m*/*z*: calcd for C_40_H_52_N_4_Cu, 651.3488, found, 651.3472 (M^+^). Single crystals suitable for X-ray diffraction were grown from a vapor diffusion of cyclohexane into a chloroform solution.

## Results and Discussion

### Synthesis of 5,10,15,20-Tetrapentylporphyrin (H_2_TPeP)

Reaction conditions for the two-step, one-flask synthesis of meso-alkyl-substituted porphyrins differ from the conditions used to prepare the more widely studied meso-aryl-substituted porphyrins. Acid catalysts (e.g., TFA, BF_3_^•^OEt_2_) that afford good yields of meso-tetraarylporphyrins generally provide lower yields of meso-tetraalkylporphyrins.^27,55^ Onaka and coworkers found that acidic clays (e.g., montmorillonite K10) give improved yields of meso-tetraalkylporphyrins,^56,57^ and these conditions have been adapted by others.^58^ H_2_TPeP in this study was prepared similarly via a two-step, one-flask reaction of pyrrole and hexanal mediated by montmorillonite K10, followed by oxidation with DDQ (Scheme 1). The reaction was performed three times on an 8.00 mmol scale. After chromatography and crystallization, H_2_TPeP was isolated in yields of 39–43% (464–510 mg).

**Scheme 1:**
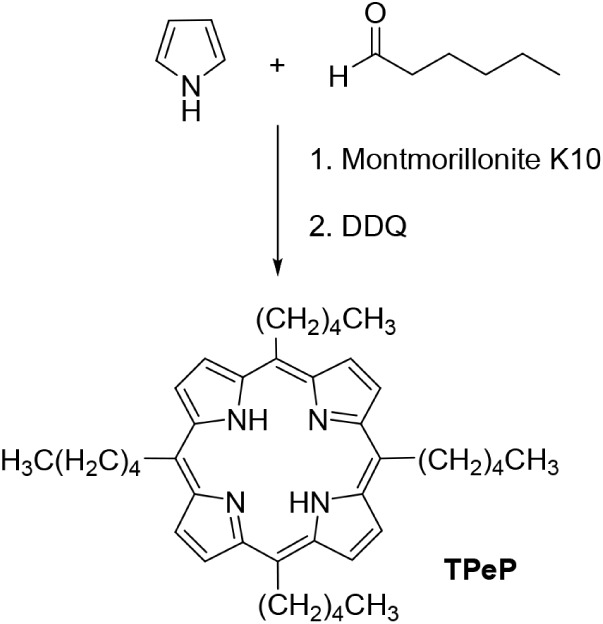
Synthesis of H2TPeP

The metalation of H_2_TPeP with copper and zinc ions is easily accomplished using standard methods, and analytically pure material can be obtained by the use of column chromatography.^59^ The compound readily forms crystals under diffusion growth conditions, and we were able to determine the structure of Cu­(TPeP) by single-crystal X-ray methods, as shown in Figure 2. To date, two structures of tetrapentylporphyrins have been elucidated: the free-base H_2_TPeP and the metalated variant Ni­(TPeP).^60,61^ The structures of the free-base H_2_TPeP and Cu­(TPeP) exhibit similar features; both are largely planar and have their pentyl groups oriented toward opposite sides of the porphyrin ring in a syn fashion. In contrast, the Ni­(TPeP) porphyrin ring is saddled, while the pentyl rings alternate between being coplanar and nearly orthogonal to the macrocycle. Ni­(OEP) is known to have three crystal forms: two planar triclinic forms (A and B) and a nonplanar tetragonal form.^62–68^ Spectroscopic investigations into solution-phase Ni­(OEP) support a planar structure. In spite of the limited crystallographic data on TPeP systems, we believe that the free base, Zn­(II), and Cu­(II) variants are all planar in solution due to the absence of steric or electronic factors that would induce nonplanarity.

**Figure 2:**
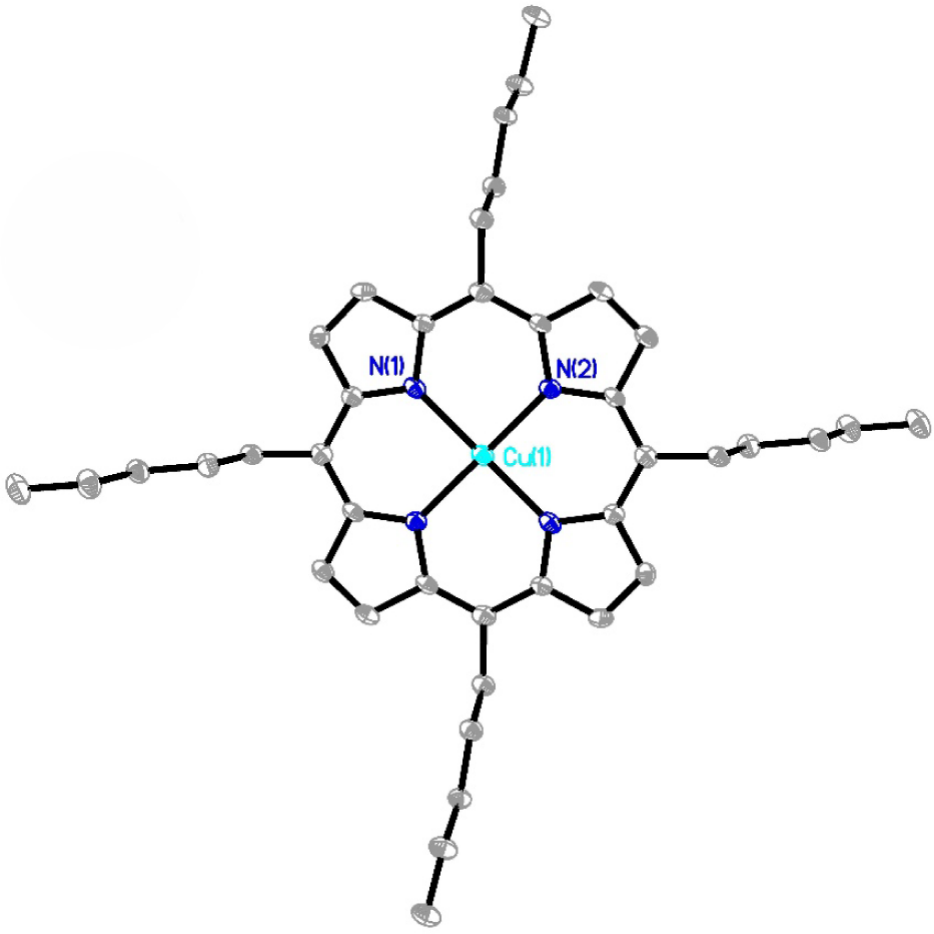
Structure of Cu­(TPeP) with 35% thermal ellipsoids. Hydrogen atom positions and a solvent cyclohexane have been omitted for clarity.

The UV–visible and MCD spectra of the H_2_TPeP and CuTPeP are shown in Figure 3, while the spectra for ZnTPeP are given in Figure 4 in comparison with those recorded for ZnTPP and ZnOEP complexes. In the case of H_2_TPeP, the Q-band region is dominated by absorption bands centered at 656, 597, 551, 517, and 483 nm, with shoulders observed at 643, 608, and 512 nm. Similar to other 5,10,15,20-tetrasubstituted porphyrins,^69–71^ the intensity of the bands in the Q-band region is about an order of magnitude lower compared with the Soret band observed at 416 nm.

**Figure 3:**
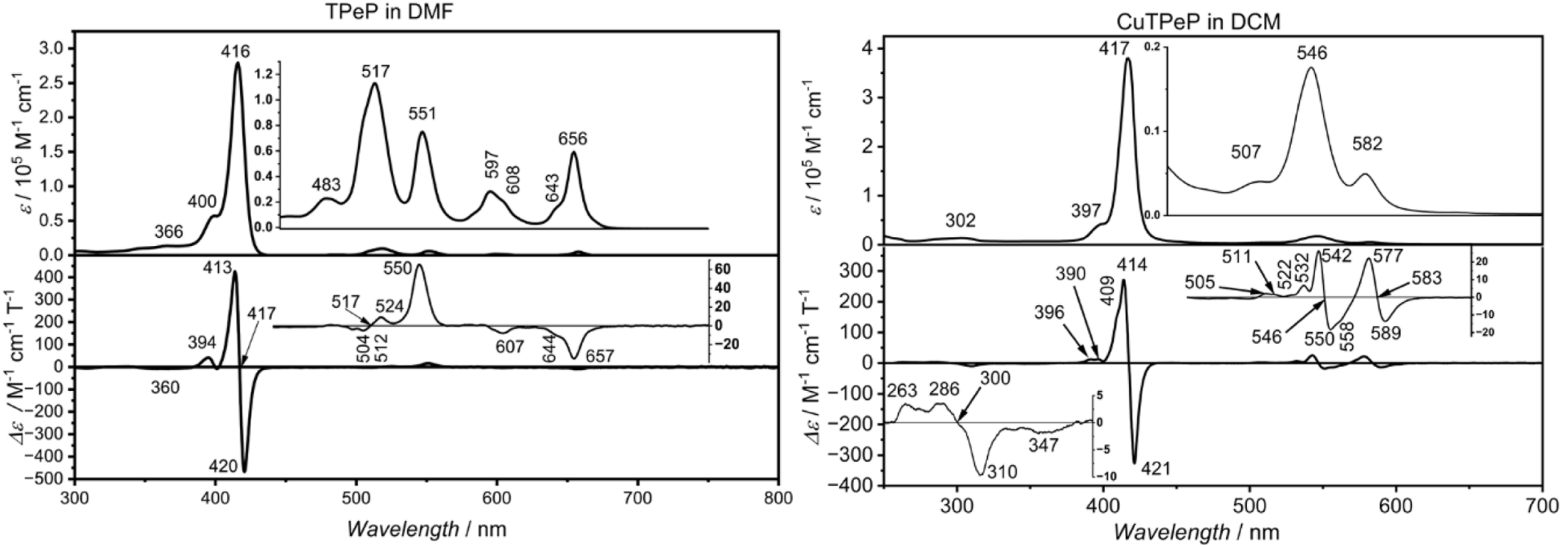
Experimental UV–visible and MCD spectra of H_2_TPeP in DMF (left) and CuTPeP in DCM (right).

**Figure 4:**
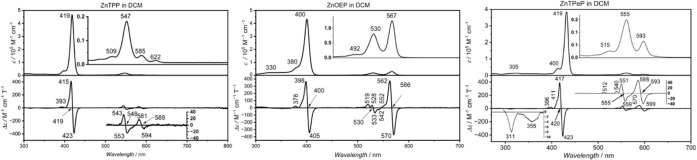
Experimental UV–visible and MCD spectra of ZnTPP (left), ZnOEP (middle), and ZnTPeP (right) in DCM.

As expected for the metal-free porphyrinoids with effective *D*_*2h*_ symmetry,^72–74^ the MCD spectra of the H_2_TPeP in the Q-band region are represented by the Faraday B-terms, which allow clear differentiation between Q_*x*_(0–0) transition observed at 657 nm and Q_*y*_(0–0) transition observed at 550 nm. Absorption bands observed at 644 and 607 nm can be clearly attributed to the vibronic satellites of the Q_*x*_ band, and the band observed at 524 nm to the vibronic satellite of the Q_*y*_ band. An increase in the effective symmetry to *D*_*4h*_ in CuTPeP and ZnTPeP complexes can be clearly seen from the appearance of the MCD Faraday A-terms observed for a doubly degenerate Q(0–0)-band at 583 and 593 nm, respectively. The Q(0–1) transitions are represented by another pair of the MCD A-terms centered at 546 and 555 nm for copper and zinc complexes, respectively. Comparison of the MCD spectra of ZnTPeP, ZnTPP, and ZnOEP complexes allows the first guess on the nature of the HOMO orbital in the ZnTPeP compound. Indeed, in the case of the ZnOEP complex, which is known to have the Gouterman’s *a*_*1u*_ orbital as the HOMO (here and below, we will use Gouterman’s four-orbital model^49–52^ to notate porphyrin-based HOMO, HOMO–1, LUMO, and LUMO+1 orbitals), the MCD intensities of the A-terms that represent the Q(0–0) transition at 566 nm and the Soret band at 400 nm are comparable to each other. The situation in the ZnTPP and ZnTPeP complexes is different, as in these cases, the intensities of the MCD A-terms associated with the Q(0–0) bands at 588 and 593 nm, respectively, are much lower compared to those associated with the Soret bands observed at 419 and 420 nm, respectively. As it is well-known that the HOMO in the ZnTPP complex has *a*_*2u*_ symmetry,^75,76^ we could expect the same symmetry of the HOMO for the ZnTPeP complex. Indeed, this was confirmed on the basis of the DFT calculations discussed below. Thus, as expected, the weak electron-donating groups in the MTPeP complexes destabilize the energy of Gouterman’s *a*_*2u*_ orbital, which has a significant density at the meso-positions of the porphyrin core.^49–52^ In general, the UV–visible spectra of the MTPeP complexes resemble those of MTPP compounds and are closely related to the spectra reported by Kadish and coworkers for the 5,10,15,20-tetraundecylporphyrins (MTUP complexes).^77^

The electrochemistry of the MTPeP compounds was studied using cyclic voltammetry (CV) and differential pulse voltammetry (DPV) methods, while the spectroscopic signatures of one-electron oxidized and one-electron reduced species were acquired with the help of the spectroelectrochemical approach. In the case of zinc and copper complexes, we observed two oxidation and one reduction process, which are similar to the processes reported earlier for MTPP, MOEP, and MTUP systems (Table 1 and Figure 5).^77^ In the case of the H_2_TPeP system, we observed two reduction and one oxidation process, with potentials close to the earlier reported H_2_TPP, H_2_OEP, and H_2_TUP compounds.^77^ However, we failed to observe a second oxidation process that was reported for these compounds. The electrochemical HOMO–LUMO gap (2.1, 2.21, and 2.11 V for zinc, copper, and metal-free compounds) is close to the expected 2.2 V gap for simple porphyrins established by Kadish.^78^ As one can see from Table 1, the MTPeP systems are the easiest to oxidize.

**Table 1:** Half-Wave Potentials (V vs SCE) of Investigated MTPeP Complexes and Related MTPP, MOEP, and MTUP Derivatives in Dichloromethane Containing 0.1 M TBAP^a^

	2nd Reduction	1st Reduction	1st Oxidation	2nd Oxidation
H_2_TPP	–1.69	–1.23	1.00	1.25
H_2_OEP	–1.89	–1.46	0.81	1.30
H_2_TUP	–1.69	–1.28	0.85	1.26
H_2_TPeP	–1.72	–1.30	0.81	–
H_2_TPeP^b^	–1.66	–1.25	0.89	–
ZnTPP	–	–1.33	0.82	1.14
ZnOEP	–	–1.61	0.63	1.02
ZnTUP	–	–1.46	0.64	1.01
ZnTPeP	–	–1.50	0.60	1.04
CuTPP	–1.72	–1.29	1.03	1.27
CuOEP	–	–1.52	0.80	1.29
CuTUP	–	–1.42	0.82	1.19
CuTPeP	–	–1.42	0.79	1.17

a MTUP, MOEP, and MTPP potentials were taken from ref 77.

b This complex was collected in dimethylformamide 0.1 M TBAPF_6_.

**Figure 5:**
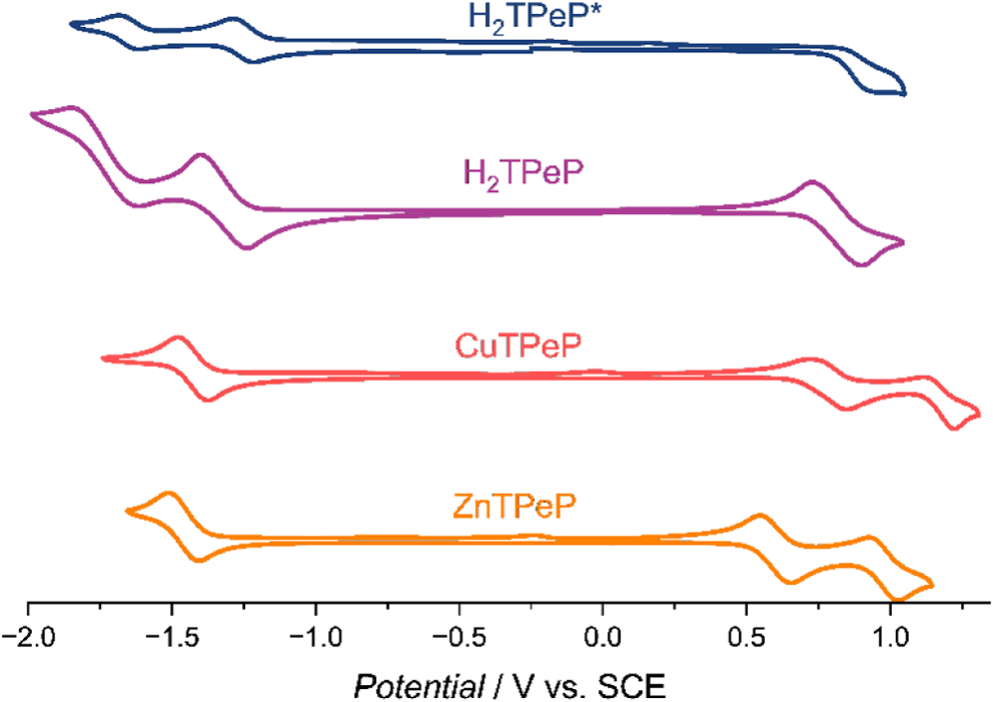
Cyclic voltammograms of MTPeP derivatives in dichloromethane 0.1 M TBAP. *This derivative was collected in dimethylformamide 0.1 M TBAPF_6_.

In the case of the ZnTPeP and CuTPeP complexes, the first oxidation studied under spectroelectrochemical conditions is clearly indicative of the oxidation of the porphyrin core (Figure 6). Indeed, in both cases, the Soret band lost its intensity and underwent a blue shift to 407 nm. This change is accompanied by the rise of the characteristic porphyrin radical band at 818 nm for ZnTPeP and 870 nm for CuTPeP complexes.^77,79,80^ The first oxidation in both systems was found to be reversible (Figure S1). During the second oxidation in ZnTPeP and CuTPeP systems, the Soret band almost completely disappears, while the NIR transition intensities continue to increase, Figure 7. However, we find that the second oxidation in both systems is only partially reversible.

**Figure 6:**
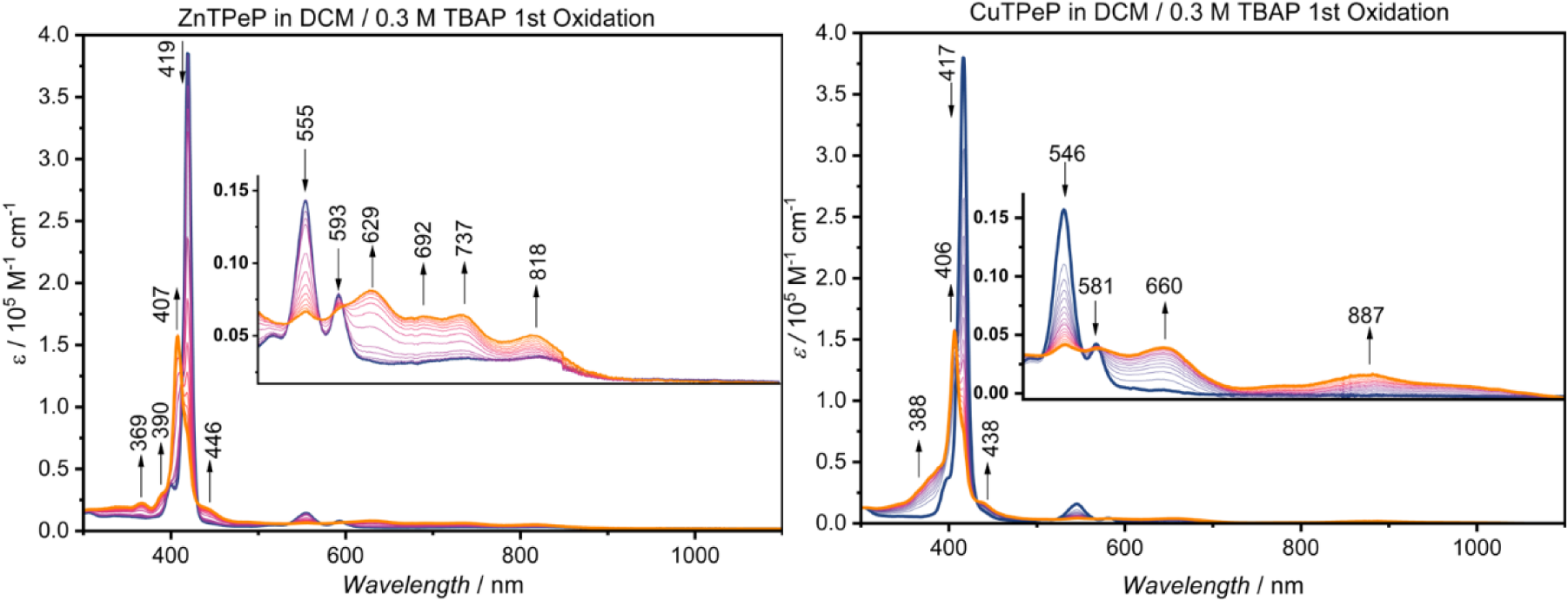
Spectroelectrochemical spectra of the 1st oxidation process of ZnTPeP and CuTPeP in dichloromethane 0.3 M TBAP.

**Figure 7:**
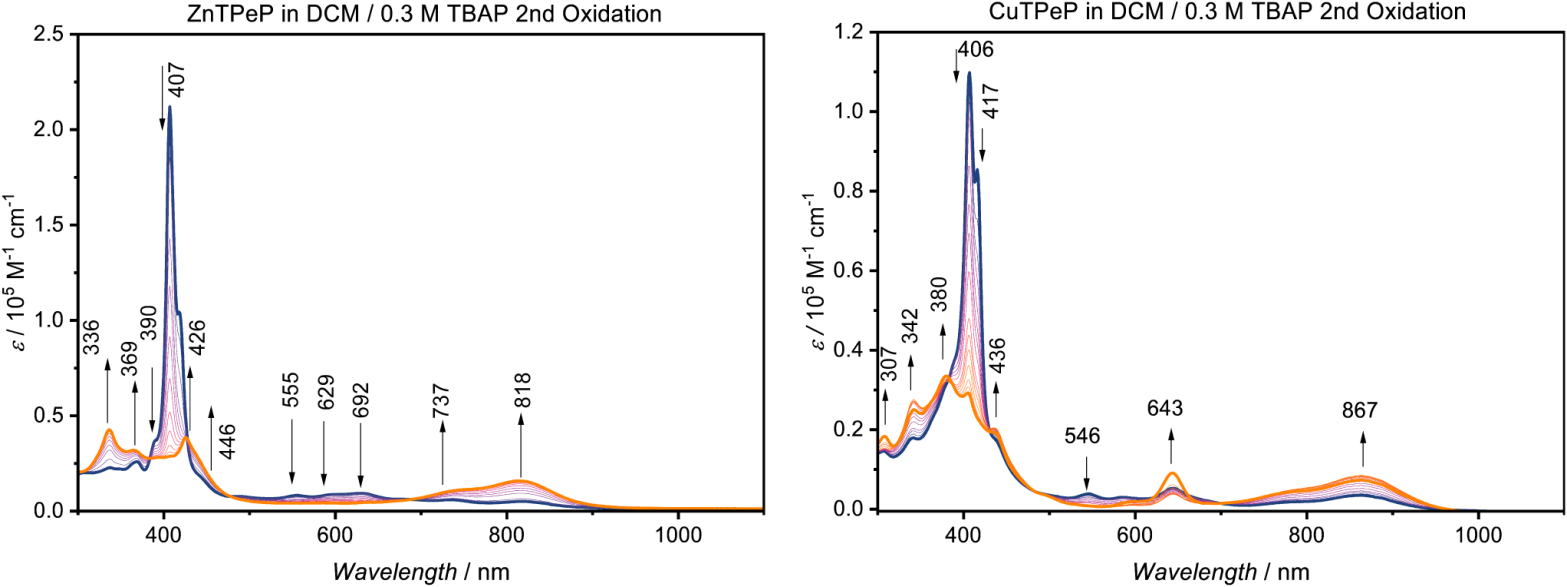
Spectroelectrochemical spectra of the 2nd oxidation process of ZnTPeP and CuTPeP in dichloromethane 0.3 M TBAP.

During the first reduction processes in ZnTPeP and CuTPeP systems, the Soret band intensity decreases, and the formation of new bands at 436 and 455 nm was observed for the ZnTPeP and CuTPeP complexes, respectively. In addition, several new bands in the Q-band region have been observed upon the reduction process, with the lowest-energy bands located at approximately 680 nm (ZnTPeP) and 820 nm (CuTPeP). The observations correlate well with earlier reports from Kadish and Crossley on MTUP complexes and are indicative of the formation of porphyrin-centered anionic radical species.^77^ Similar to the metalated systems, the first reduction of the metal-free H_2_TPeP complex system leads to the formation of the anionic radical with characteristic bands at 450, 802, and 900 nm (Figure 8).

**Figure 8:**
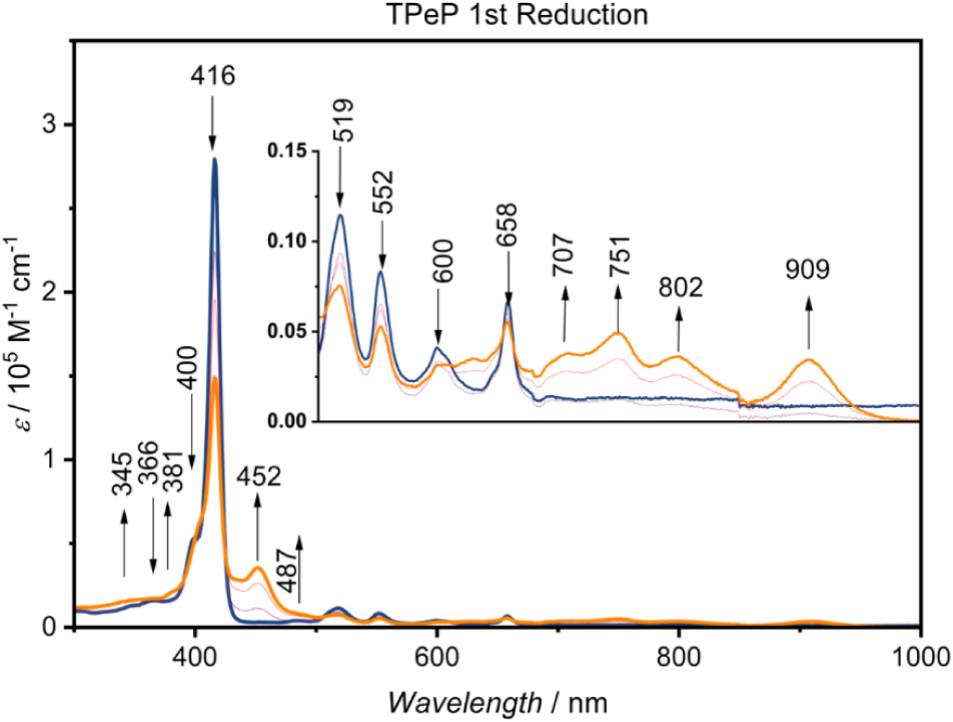
Spectroelectrochemical spectra of the 1st reduction process of H_2_TPeP in dimethylformamide 0.3 M TBAPF_6_.

The oxidation of the free-base system, however, is completely different. During the first oxidation process, the Soret band in the metal-free H_2_TPeP complex undergoes a red shift from 418 to 425 nm, and the intensity of the new Soret band is comparable to the intensity of the initial one. In addition, the Q_*x*_ band observed at 659 nm undergoes a blue-shift to 635 nm with an intensity increase. These changes correlate well with the formation of a doubly protonated neutral [H_4_TPeP]^2+^ core. Indeed, in a separate experiment, when trifluoroacetic acid was titrated into a solution of the H_2_TPeP, exactly the same changes were observed in the UV–visible spectra (Figure 9), with the protonated species containing a 4-fold effective symmetry, which can be clearly seen in its MCD spectra (Figure 9). Both the Soret and Q(0–0) bands are associated with the MCD-A terms observed at 421 and 631 nm, respectively. The protonation of the metal-free porphyrin during its oxidation under spectroelectrochemical conditions is not new; it has been previously observed.^77,81^

**Figure 9:**
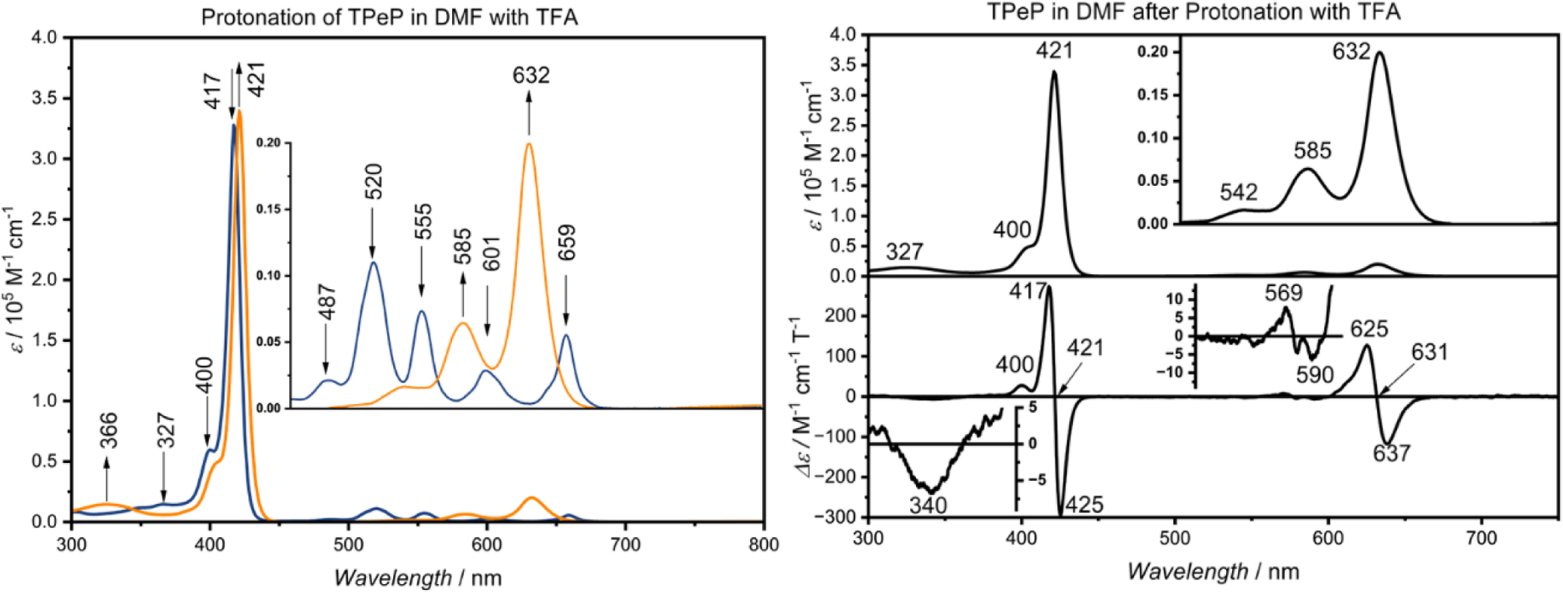
UV–visible spectra of H_2_TPeP before and after the addition of trifluoroacetic acid (left). UV–visible and MCD spectra of [H_4_TPeP]^2+^ in dimethylformamide (right).

In order to elucidate the electronic structures and nature of the excited states in the MTPeP compounds, we have conducted a series of DFT and TDDFT calculations on the neutral diamagnetic ZnTPeP and H_2_TPeP along with their redox-active or protonated counterparts. The energy diagrams of the compounds of interest are shown in Figure 10, with selected frontier orbitals shown in Figure 11 and SI Figures S2–S9. Finally, the TDDFT spectra of the compounds of interest are compared to the experimental data in Figures 12 and 13. A color-coded diagram of the transitions in ZnTPeP and H_2_TPeP and their corresponding radical anions and cations, is shown in Figure 14.

**Figure 10:**
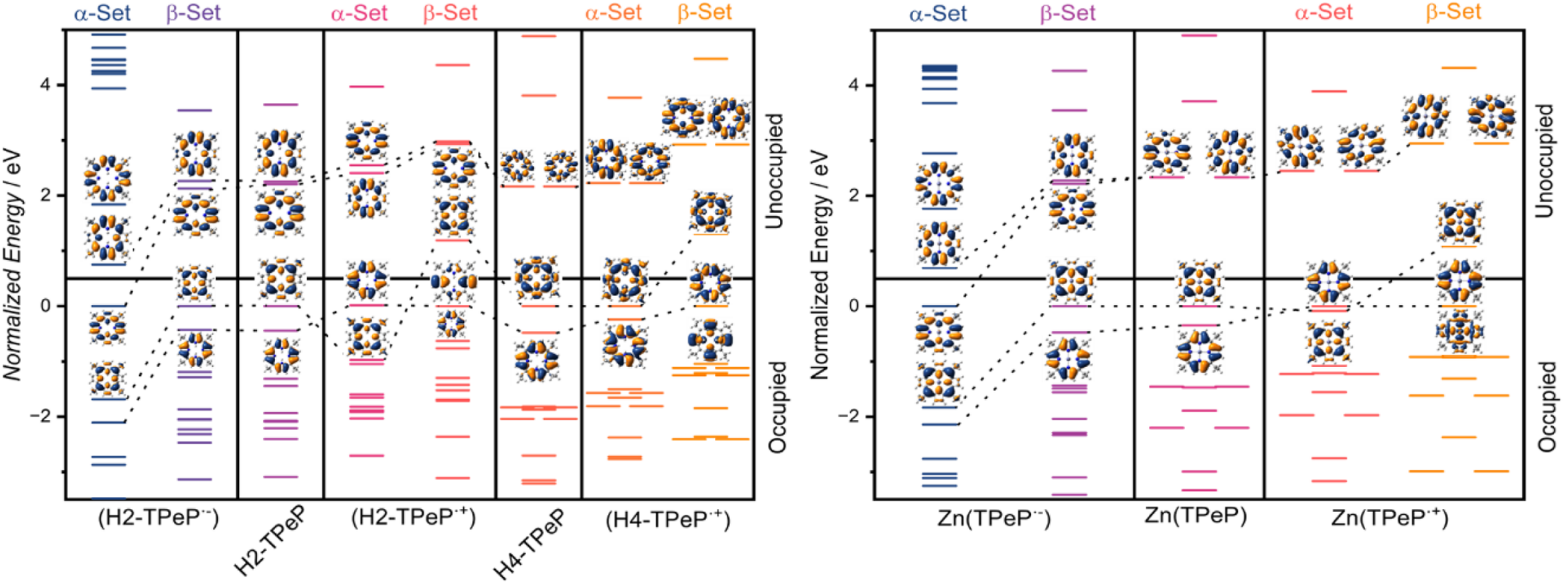
DFT-predicted energy diagrams of H_2_TPeP, [H_4_TPeP]^2+^, and ZnTPeP complexes.

**Figure 11:**
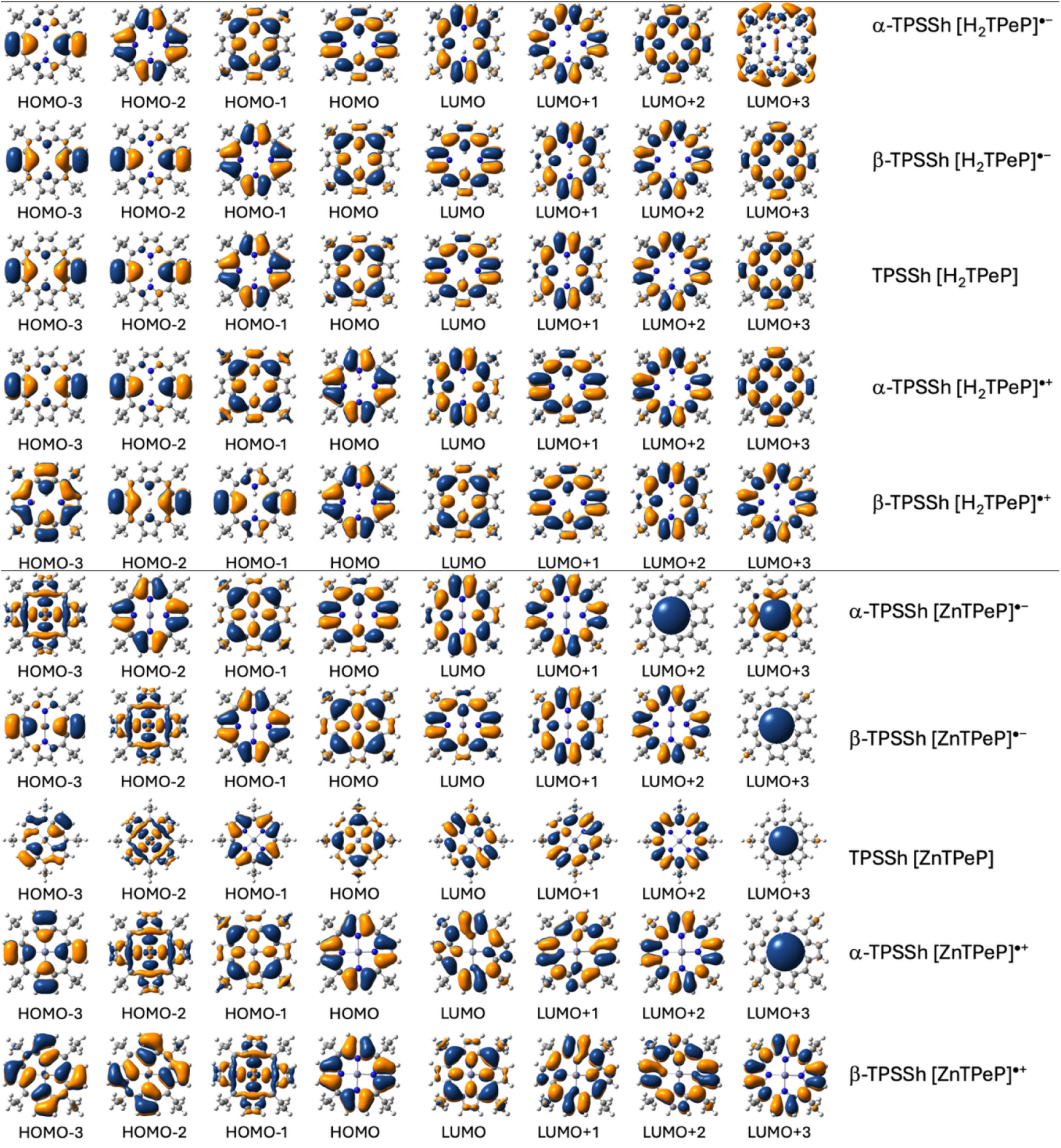
Select DFT-calculated frontier orbital images of H_2_TPeP, ZnTPeP, and their redox-active derivatives.

**Figure 12:**
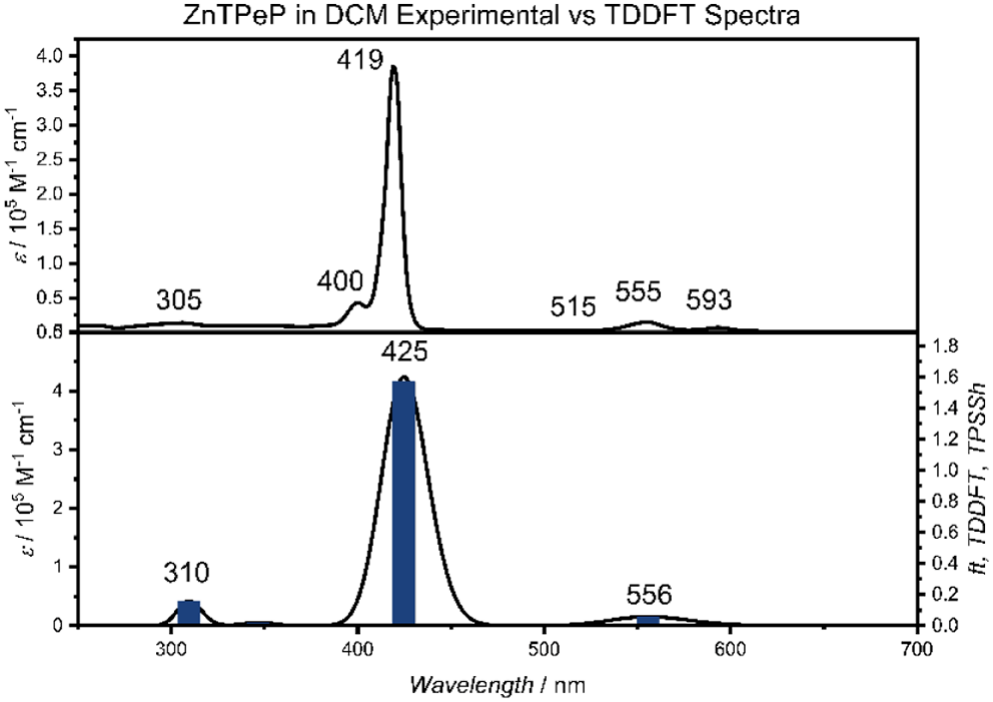
Experimental vs TDDFT-predicted UV–visible spectra of the neutral ZnTPeP species in dichloromethane.

**Figure 13:**
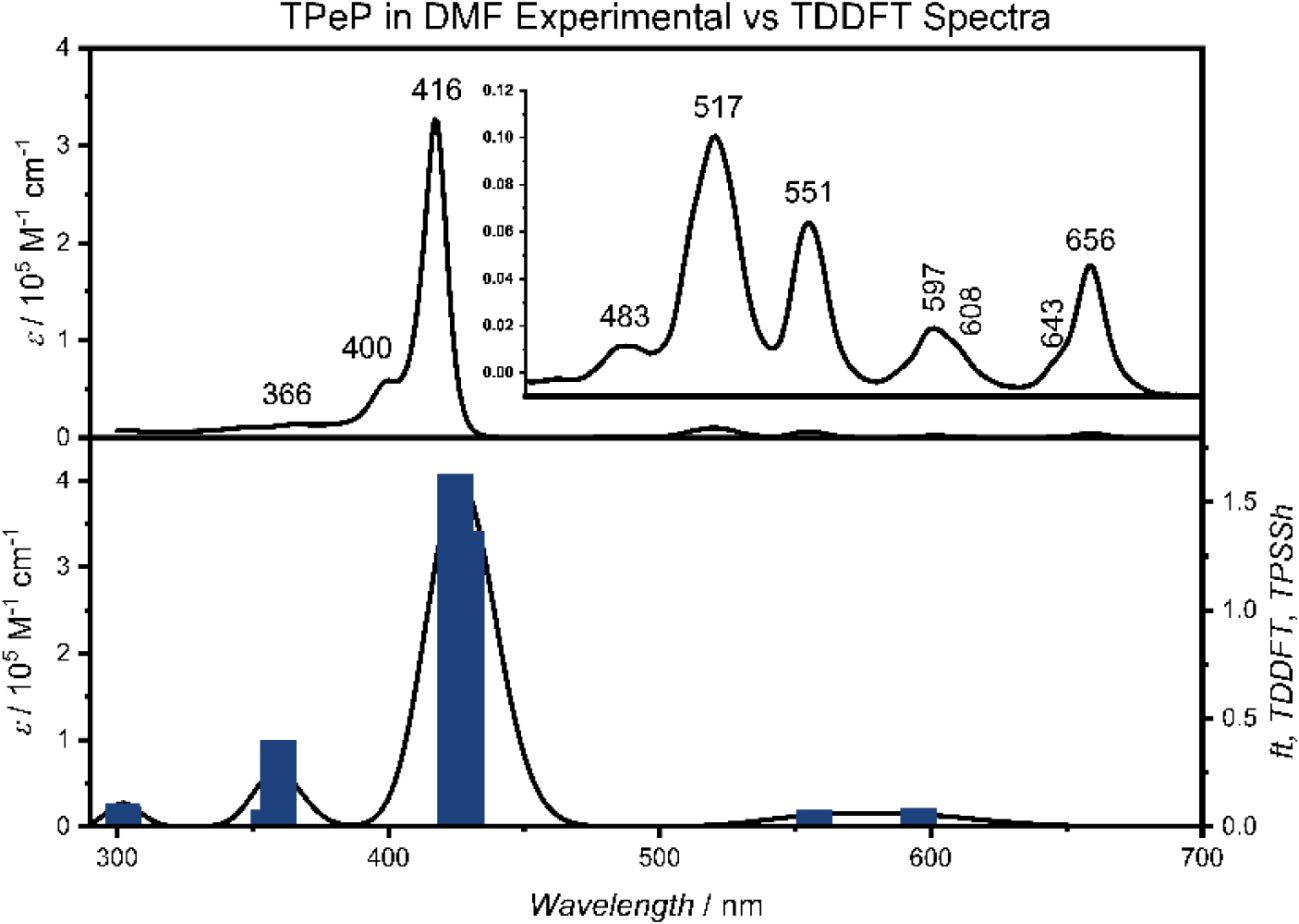
Experimental vs TDDFT-predicted UV–visible spectra of the neutral H_2_TPeP species in dichloromethane.

**Figure 14:**
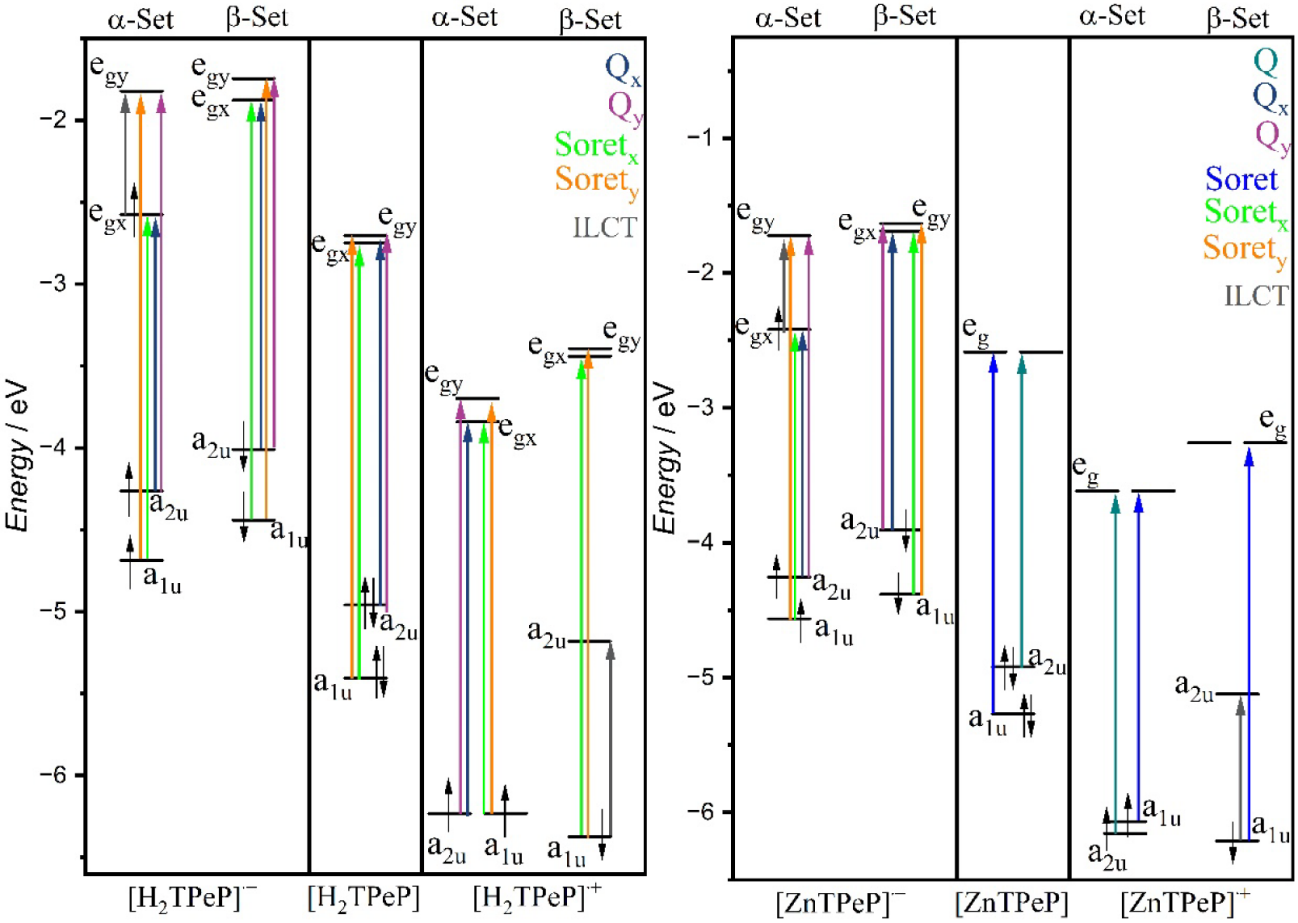
Schematic diagrams of the optical transitions in ZnTPeP and H_2_TPeP and their radical cations and anions.

In the case of the neutral ZnTPeP complex, the HOMO and HOMO–1 orbitals, as Gouterman’s *a*_*2u*_ and *a*_*1u*_ symmetry, were predicted on the basis of its MCD spectrum, and this is also the case for the metal-free H_2_TPeP compound. The LUMO and LUMO+1 orbitals in the ZnTPeP and metal-free H_2_TPeP systems also belong to Gouterman’s pair of *e*_*gx*_ and e_*gy*_ orbitals. The splitting between the pair of Gouterman’s *e*_*g*_ orbitals in the metal-free compound is rather small (here and below, we will keep “*e*_*gx*_” label for *b*_*2g*_ and “*e*_*gy*_” label for *b*_*3g*_” MOs in order to stay within Gouterman’s four-orbital labeling). In the case of the ZnTPeP complex, the TDDFT predicts a degenerate Q-band at 556 nm and a Soret band at 425 nm, which correlate well with the experimental spectrum of this compound, with the TDDFT-predicted Q-band and Soret band energies falling within the range expected for TDDFT calculations. In the case of the metal-free H_2_TPeP compound, the geometry converged to *D*_2_ symmetry, and thus, the degeneracy of the Q-band and Soret band is removed. TDDFT predicted the Q_*x*_ and Q_*y*_ splitting to be larger compared to the Soret_*x*_ and Soret_*y*_, which agrees with experimental data. The symmetry of the doubly protonated [H_4_TPeP]^2+^ compound was found to be S_4_ and thus the Q_*x*_ and Q_*y*_ as well as the Soret_*x*_ and Soret_*y*_ transitions, become doubly degenerate again, in agreement with experimental data. The TDDFT-predicted energies of the Q-band and Soret band in the spectra are also within the range of error expected for TDDFT-predicted calculations. Overall, as expected, the spectroscopy of the neutral MTPeP compounds follows the classic Gouterman’s model.^49–52^ In agreement with the spectroelectrochemical data, the formation of the porphyrin-centered cation radical upon oxidation of ZnTPeP complex should give arise of the NIR band around 800 nm. Indeed TDDFT predicted such a band, which is dominated by a single-electron transition from the filled *e*_*g*_ symmetry orbital to he half-filled *a*_*2u*_ orbital in the [ZnTPeP]^+.^ radical complex at 792 nm, correlating well with the NIR band experimentally observed at 818 nm. The blue shift of the Soret band upon oxidation of the neutral ZnTPeP is also well predicted by the TDDFT calculations (Figure 14). Additionally, in agreement with the experiment data, the set of new bands between 480 and 600 nm is also predicted by TDDFT. Many of these bands have a significant contribution from the single-electron excitation to the half-filled *a*_*2u*_ orbital (Table S11). Overall, the agreement between the theoretical spectra and the experimental spectra of oxidized [ZnTPeP]^+.^ complexes is rather good (Figure 15).

**Figure 15:**
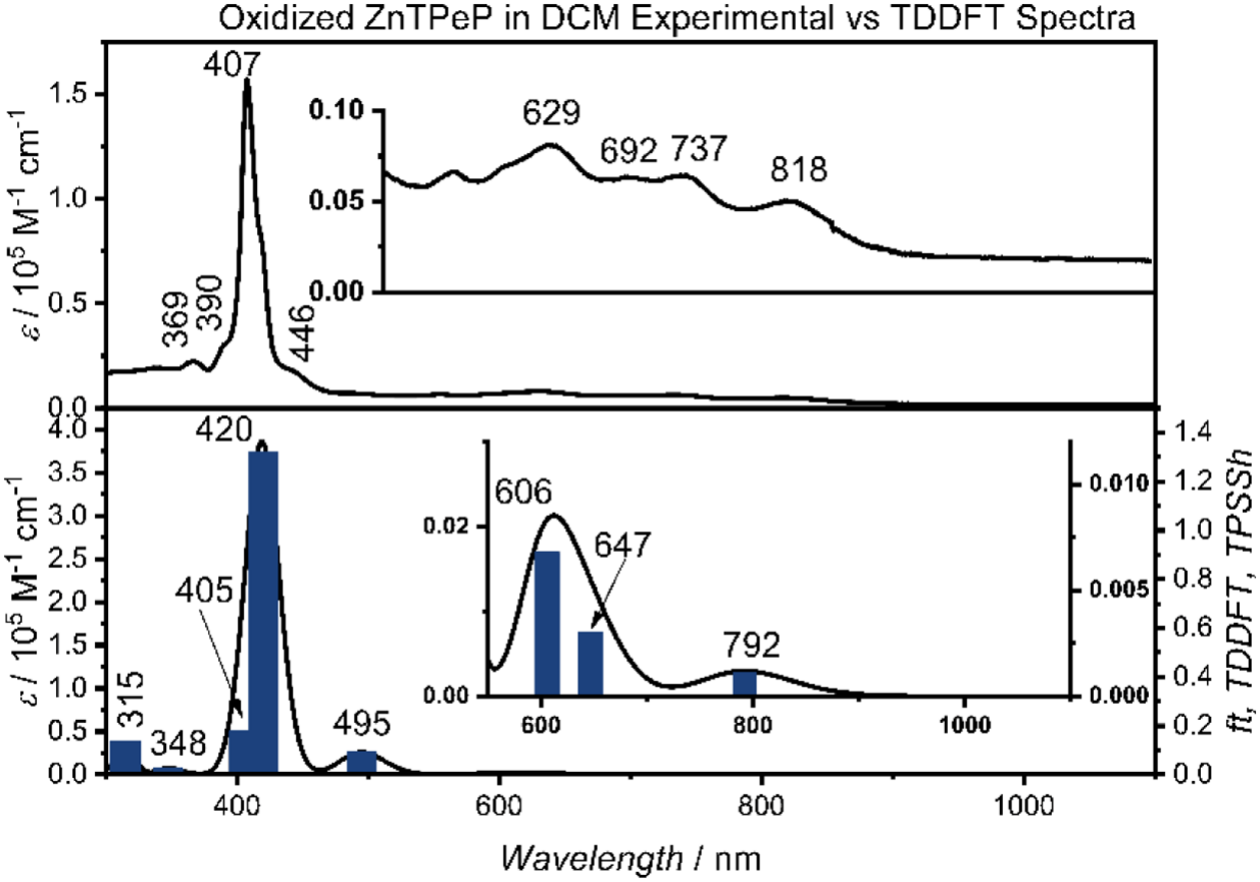
Experimental vs TDDFT-predicted UV–visible spectra of the [ZnTPeP]^•+^ species in dichloromethane.

Adding an extra electron to the neutral ZnTPeP complex leads to the doubly degenerate ^2^ *E*_*g*_ ground state term, which is similar to other porphyrin anion radicals and is the subject of Jahn–Teller distortion. Indeed, DFT predicted the geometry for the [ZnTPeP]^−.^ anion-radical complex to have *D*_2_ symmetry. The same geometry point group symmetry was also predicted for the metal-free H_2_TPeP compound. For [ZnTPeP]^−.^ and metal-free [H_2_TPeP]^−.^ anion-radical complexes, TDDFT predicts the presence of NIR transitions at around 952 nm. This transition is dominated by single-electron excitations from the occupied Gouterman’s *a*_*2u*_ orbital to the empty “*e*_*gy*_“molecular orbital; as well as single-electron excitations from the half-filled “*e*_*gx*_“orbital to the unoccupied “*e*_*gy*_“orbital. In addition, a number of excited states between 500 and 800 nm were predicted by TDDFT calculations (Table S11) many of these involve excitations to the half-filled or unoccupied Gouterman’s *e*_*gx*_ and *e*_*gy*_ pair of orbitals. TDDFT also correctly predicts the red-shifted Soret band in metal-free H_2_TPeP and ZnTPeP systems. Overall, TDDFT predictions correlate well with the experimental data, not only for the neutral species but also for the redox-active species of the meso-substituted porphyrin.

## Conclusions

In conclusion, H_2_TPeP, Zn­(TPeP), and Cu­(TPeP) have been investigated by spectroscopic, electrochemical, and computational methods, and their properties have been compared to the corresponding TPP, OEP, and TUP systems. The four orbital structures of free base and metalated TPeP resemble those of the meso-substituted TPP, with an inverted HOMO/HOMO–1 relative to the corresponding OEP compounds. The three TPeP compounds are also easier to reduce than OEP or TPP systems and, not surprisingly, have redox properties similar to those observed in TUP. DFT and TDDFT methods helped elucidate the electronic structures and are in good agreement with experimental data.

## Supplementary Material



## Notes

Spectroelectrochemistry data, computational results, and crystallographic data collection parameters (PDF)
