# Supplementary material for: Structure and Spectroscopy of Free Base, Copper, and Zinc Tetrapentylporphyrin
Source: J Phys Chem A. 2026 Jun 9;130(24):4594–605. doi: 10.1021/acs.jpca.6c02509 (PMC13288633; doi:10.1021/acs.jpca.6c02509)
Supplement: Supplementary file 1 [file jp6c02509_si_001.pdf]

Structure and Spectroscopy of Free Base, Copper, and Zinc Tetrapentylporphyrin

Breanna Muldowney,[a] Nneka Damola Ajayi,[b] Wei-Yuan Chen,[a] G. Richard Geier III,[c] Christopher J. Ziegler,[b*] Victor N. Nemykin[a*]

[a.] Department of Chemistry, University of Tennessee, Knoxville, TN 37996, USA
Email: ziegler@uakron.edu
[b.] Department of Chemistry, University of Akron, Akron, OH 44325, USA
Email: vnemykin@utk.edu
[c.] Department of Chemistry, Colgate University, Hamilton, NY 13346, USA

Supporting Information

Table of Contents

Figure S1. Spectroelectrochemical reductive recovery of ZnTPeP and CuTPeP after first oxidation in dichloromethane 0.3 M TBAP……………………………………………………….S3

Figure S2. Calculated molecular orbital images of H_2TPeP……………………………………S3

Figure S3. Calculated molecular orbitals of $[H_2TPeP]^{\bullet+}$………………………………………S4

Figure S4. Calculated molecular orbitals of $[H_2TPeP]^{\bullet-}$………………………………………S4

Figure S5. Calculated molecular orbital images of H_4TPeP……………………………………S5

Figure S6. Calculated molecular orbitals of $[H_4TPeP]^{\bullet+}$……………………………………….S5

Figure S7. Calculated molecular orbital images of ZnTPeP……………………………………S5

Figure S8. Calculated molecular orbital images of $[ZnTPeP]^{\bullet+}$ ……………………………….S6

Figure S9. Calculated molecular orbital images of $[ZnTPeP]^{\bullet-}$ ……………………………….S6

Table S1. TDDFT predicted energies, oscillator strengths, and contributions for the major excited states for the neutral H_2TPeP complex and its redox-active derivatives. ……………...S7

Table S2. TDDFT predicted energies, oscillator strengths, and contributions for the major excited states for the H4TPeP complex and its redox-active derivatives. ……………………..S8

Table S3. DFT-predicted molecular orbital compositions for TPeP[a]…………………………..S10

Table S4. DFT-predicted molecular orbital compositions for oxidized TPeP[a]…………………S11

Table S5. DFT-predicted molecular orbital compositions for reduced TPeP[a]………………….S12

Table S6. DFT-predicted molecular orbital compositions for protonated TPeP[a]……………….S12

Table S7. DFT-predicted molecular orbital compositions for oxidized protonated TPeP[a]……..S13

Table S8. DFT-predicted molecular orbital compositions for ZnTPeP[a]………………………..S14

Table S9. DFT-predicted molecular orbital compositions for oxidized ZnTPeP[a]……………...S14

Table S10. DFT-predicted molecular orbital compositions for reduced ZnTPeP[a] …………….S15

Table S11. TDDFT predicted energies, oscillator strengths, and contributions for the major excited states for the neutral ZnTPeP complex and its redox-active derivatives………………S16

Table S12. Crystal data and structure refinement for Cu(TpeP)……………………………...S22

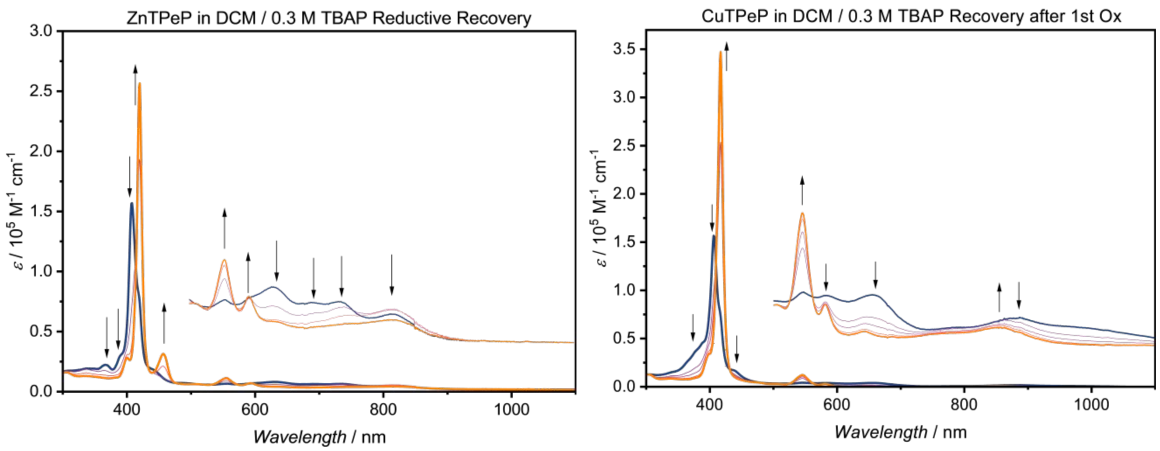

Figure S1. Spectroelectrochemical reductive recovery of ZnTPeP and CuTPeP after first oxidation in dichloromethane 0.3 M TBAP.

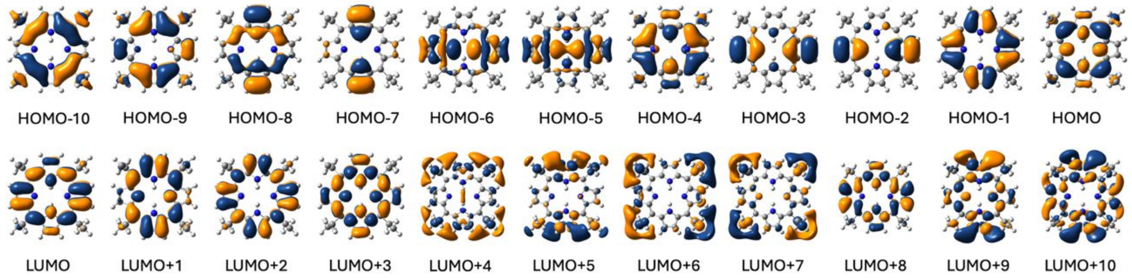

Figure S2. Calculated molecular orbital images of H_2TPeP

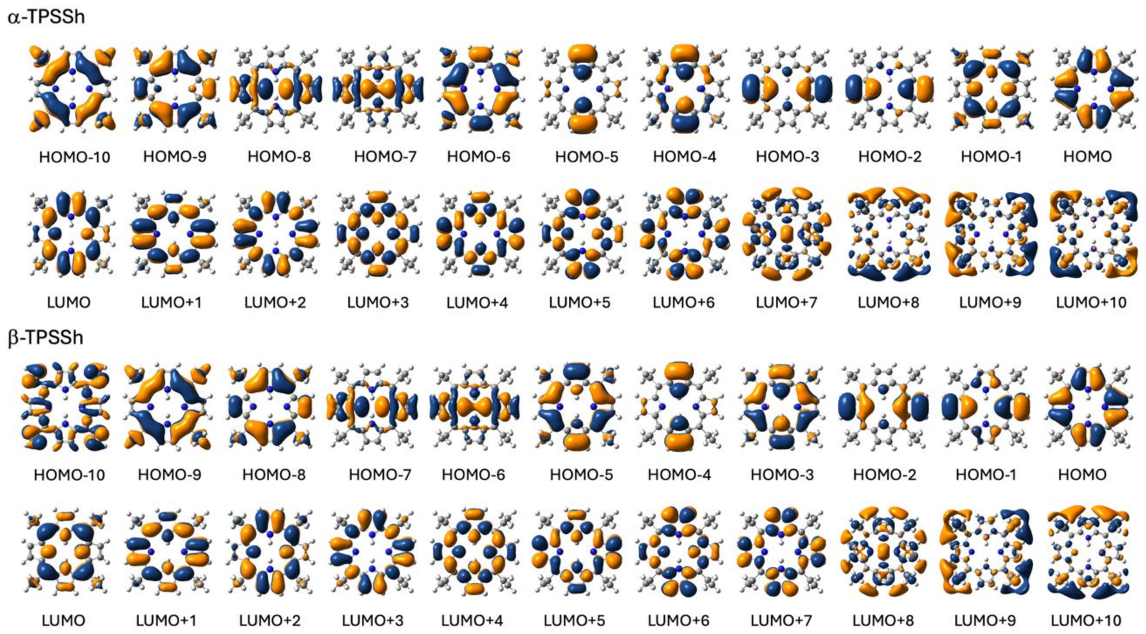

Figure S3. Calculated molecular orbitals of $[H_2TPeP]^{\bullet +}$

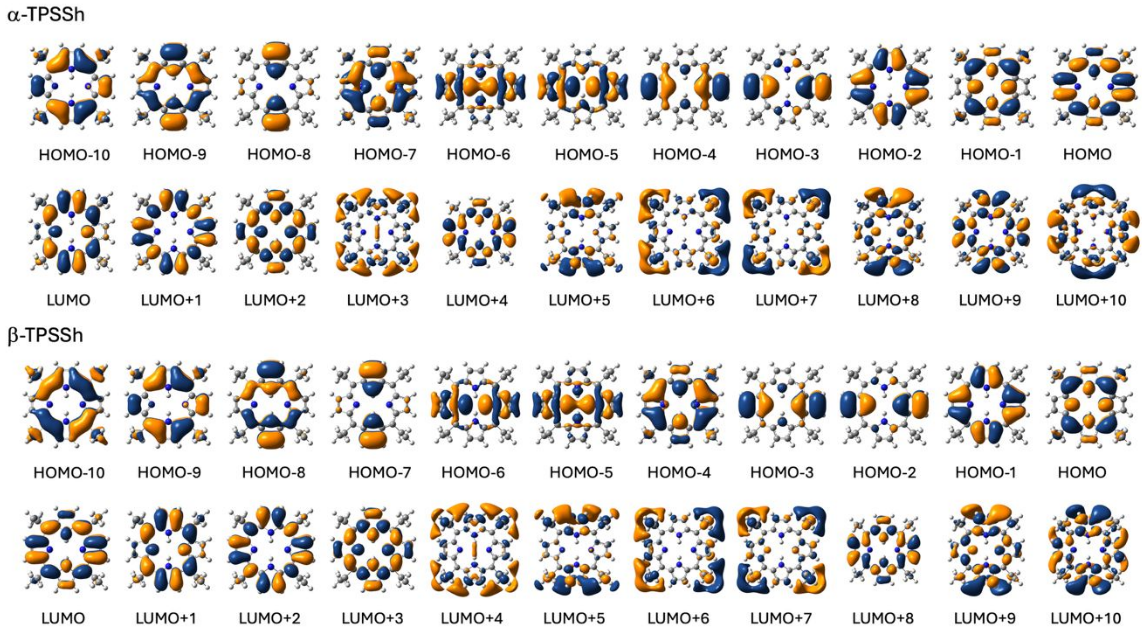

Figure S4. Calculated molecular orbitals of $[H_2TPeP]^{\bullet -}$

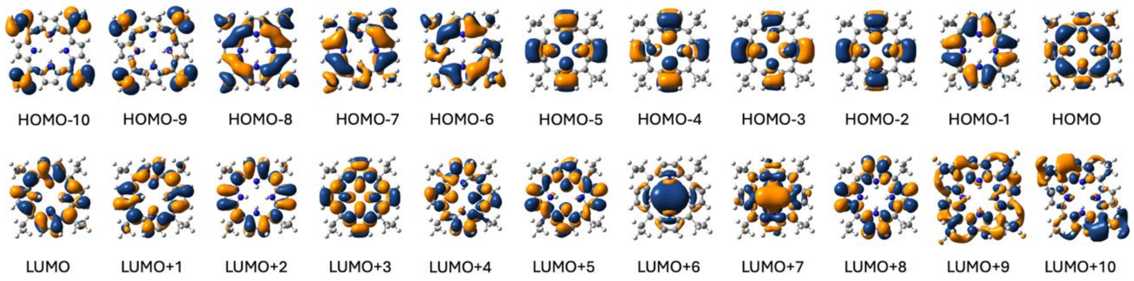

Figure S5. Calculated molecular orbital images of H_4TPeP

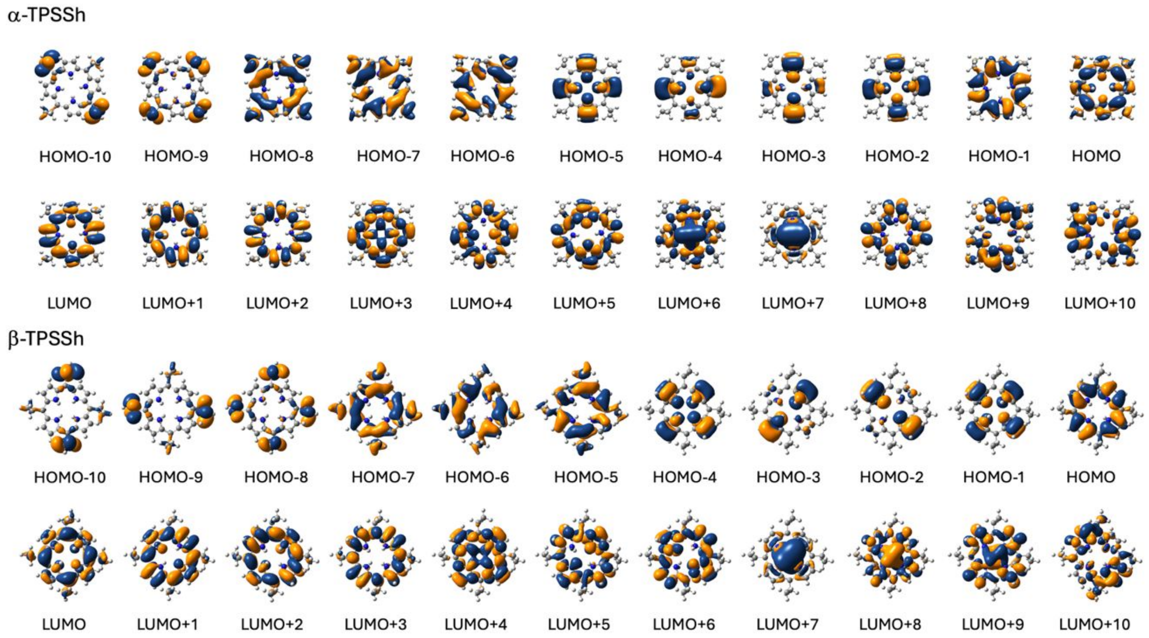

Figure S6. Calculated molecular orbitals of $[H_4TPeP]^{\bullet+}$

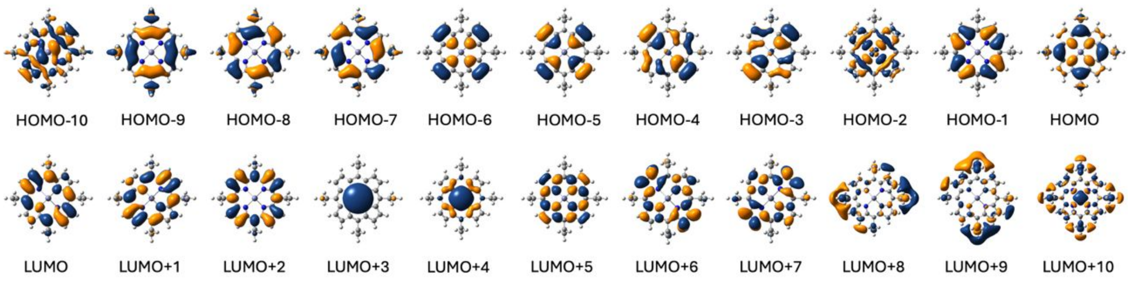

Figure S7. Calculated molecular orbital images of ZnTPeP

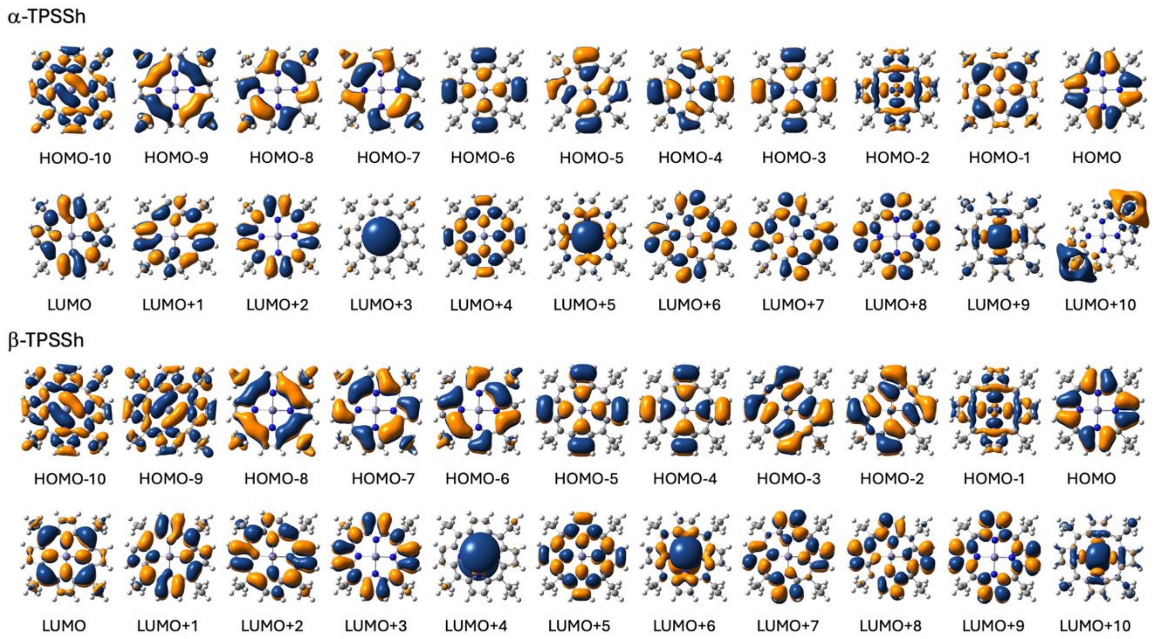

Figure S8. Calculated molecular orbital images of $[ZnTPeP]^{\bullet+}$

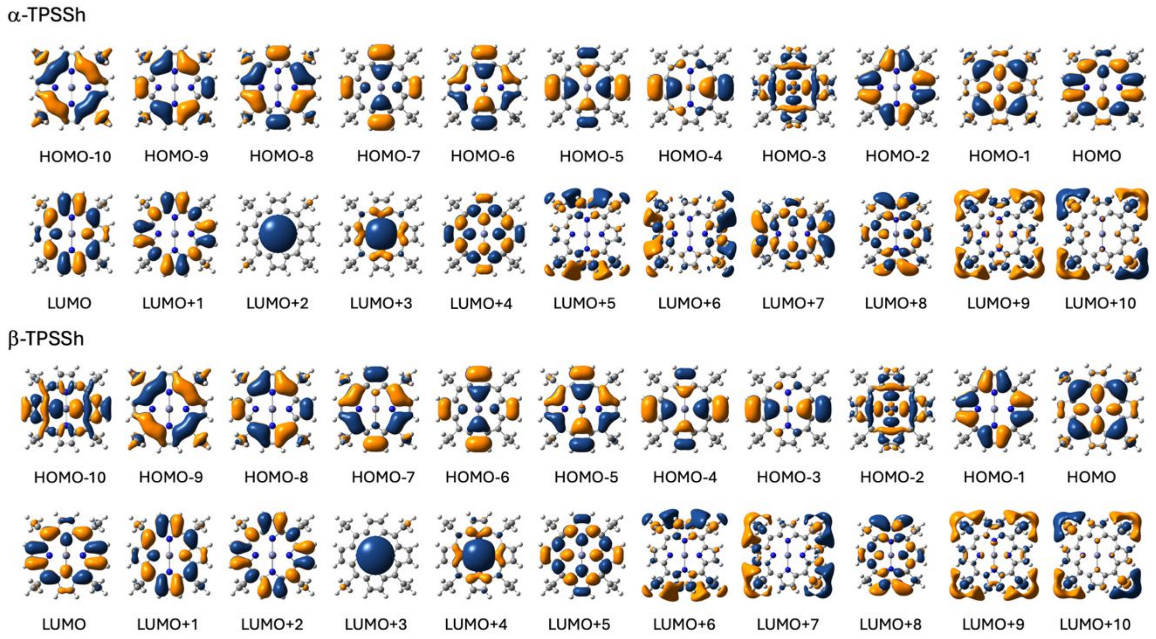

Figure S9. Calculated molecular orbital images of $[ZnTPeP]^{\bullet-}$

Table S1. TDDFT predicted energies, oscillator strengths, and contributions for the major excited states for the neutral H_2TPeP complex and its redox-active derivatives.

Excited State	Energy (cm^{-1})	Wavelength (nm)	Osc. Strength	Band Assignment	Contributions[a]
			$[H_2TPeP]$		
1	16799	595	0.0875		77.5% HOMO → LUMO, 22.1% H-1 → L+1
2	17954	557	0.0799		72.5% HOMO → L+1, 27.3% H-1 → LUMO
3	23326	429	1.3600		75.2% H-1 → L+1, 20.7% HOMO → LUMO, 4.94% H-3 → LUMO
4	23557	425	1.6300		72.5% H-1 → LUMO, 28.2% HOMO → L+1
7	27816	360	0.4010		94.2% H-3 → LUMO, 2.82% H-1 → L+1, 2.65% HOMO → LUMO
8	28063	356	0.0001		94.1% HOMO → L+2, 2.54% H-8 → L+1
9	28108	356	0.0811		99.0% H-3 → L+1
10	31047	322	0.0002		99.5% H-5 → LUMO
12	31576	317	0.0003		74.4% H-4 → L+1, 21.9% H-7 → L+1, 2.03% H-9 → LUMO
14	31733	315	0.0001		99.9% H-5 → L+1
15	32027	312	0.0014		77.7% H-7 → L+1, 19.6% H-4 → L+1
17	33069	302	0.0954		99.0% H-6 → LUMO
18	331134	302	0.1060		98.8% H-6 → L+1
			$[H_2TPeP]^{\bullet+}$		
2	11165	896	0.0035		89.6% H-1(β) → LUMO(β)
4	14895	671	0.0120		48.2% HOMO(α) → L+1(α), 36.9% H-1(α) → LUMO(α)
5	15208	658	0.0008		48.7% H-1(α) → L+1(α), 42.2% H-3(β) → LUMO(β)
6	15465	647	0.0133		78.3% HOMO(α) → LUMO(α), 11.3% HOMO(β) → L+2(β)
7	16807	595	0.0529		34.3% H-1(α) → LUMO(α), 23.0% HOMO(α) → L+1(α), 19.8% H-8(β) → LUMO(β), 18.5% HOMO(β) → L+1(β)
8	17916	558	0.0011		42.5% H-3(β) → LUMO(β), 38.0% H-5(β) → LUMO(β), 15.5% H-1(α) → L+1(α)
10	18757	533	0.0004		98.2% H-6(β) → LUMO(β)
12	20053	499	0.1130		39.3% H-5(β) → LUMO(β), 34.5% HOMO(β) → L+2(β), 18.8% H-1(α) → L+1(α)
13	20899	478	0.0337		58.4% H-8(β) → LUMO(β), 30.2% HOMO(β) → L+1(β)
15	22754	439	0.0159		51.7% H-3(α) → L+1(α), 35.6% H-2(β) → L+1(β)
17	23780	421	0.0002		63.2% H-9(β) → LUMO(β), 17.4% H-1(α) → L+2(α)
18	23947	418	1.3800		50.4% HOMO(β) → L+2(β), 18.3% HOMO(α) → LUMO(α), 13.5% H-1(α) → L+1(α)
19	23994	417	1.0800		31.3% HOMO(β) → L+1(β), 23.6% H-3(α) → LUMO(α), 21.4% HOMO(α) → L+1(α)
20	24744	404	0.5390		57.6% H-3(α) → LUMO(α), 13.5% H-2(β) → L+2(β), 10.6% HOMO(β) → L+1(β)
25	27458	364	0.0001		67.0% H-5(α) → LUMO(α), 19.2% H-4(β) → L+2(β)
26	27699	361	0.0859		79.6% H-2(β) → L+2(β), 14.6% H-3(α) → LUMO(α)
27	27773	360	0.4530		55.8% H-2(β) → L+1(β), 36.8% H-3(α) → L+1(α)
29	29152	343	0.0001		50.0% H-1(α) → L+2(α), 28.2% H-9(β) → LUMO(β)
30	30218	331	0.0031		66.0% H-5(α) → L+1(α), 28.8% H-4(β) → L+1(β)
34	30966	323	0.0004		90.8% H-8(α) → LUMO(α)
40	32129	311	0.0001		34.4% H-8(β) → L+1(β), 28.0% H-9(α) → L+1(α), 14.0% H-3(β) → L+2(β)
42	32411	309	0.0089		92.7% H-12(β) → LUMO(β)
43	32528	307	0.0812		72.4% H-4(β) → L+2(β), 24.7% H-5(α) → LUMO(α)
45	33004	303	0.0730		67.6% H-4(β) → L+1(β), 30.6% H-5(α) → L+1(α)
47	33387	300	0.0103		94.8% H-13(β) → LUMO(β)
51	33883	295	0.0003		91.6% H-6(β) → L+2(β)
52	34040	294	0.0009		61.9% H-7(β) → L+2(β), 33.3% H-14(β) → LUMO(β)
53	34065	294	0.0010		94.9% H-15(β) → LUMO(β)
54	34079	293	0.0004		64.3% H-14(β) → LUMO(β), 30.4% H-7(β) → L+2(β)
55	34725	288	0.0001		96.7% H-16(β) → LUMO(β)
57	35154	284	0.0010		42.7% H-2(α) → L+2(α), 33.6% H-1(β) → L+3(β)

58	36087	277	0.0007	66.8% H-17(β) → LUMO(β), 16.2% H-3(α) → L+2(α)
59	36112	277	0.0005	33.4% H-3(α) → L+2(α), 31.1% H-17(β) → LUMO(β), 17.2% H-2(β) → L+3(β)
60	36812	272	0.0044	53.2% H-10(α) → LUMO(α), 25.5% H-9(β) → L+2(β)
				$[H_2TPeP]^{\bullet-}$
2	10503	952	0.0684	43.7% HOMO(α) => L+1(α), 43.3% HOMO(β) => L+1(β), 13.9% H-1(α) => LUMO(α)
3	13437	744	0.0468	45.9% HOMO(α) => L+1(α), 22.7% H-1(α) => LUMO(α), 19.0% HOMO(β) => L+1(β), 12.2% H-1(β) => LUMO(β)
4	14453	692	0.1090	93.0% HOMO(β) => LUMO(β)
5	17630	567	0.0063	58.0% H-1(β) => LUMO(β), 34.2% H-1(α) => LUMO(α)
6	17631	567	0.0180	61.2% H-1(β) => L+1(β), 35.6% H-2(α) => LUMO(α)
7	21643	462	1.5600	33.4% HOMO(β) => L+1(β), 28.9% H-1(β) => LUMO(β), 28.8% H-1(α) => LUMO(α)
8	22410	446	0.5160	53.5% H-2(α) => LUMO(α), 21.9% H-1(β) => L+1(β), 15.8% H-3(β) => LUMO(β)
12	23529	425	0.3560	76.5% H-3(β) => LUMO(β)
13	24784	403	0.0021	62.0% H-3(β) => L+1(β), 35.7% H-4(α) => LUMO(α)
17	27204	368	0.0810	62.6% H-4(α) => LUMO(α), 36.3% H-3(β) => L+1(β)
20	29161	343	0.6610	86.6% HOMO(α) => L+2(α)
21	29277	342	0.0225	42.8% H-7(β) => L+1(β), 39.9% H-8(α) => LUMO(α)
23	29901	334	0.0001	99.2% H-5(β) => LUMO(β)
24	29928	334	0.0001	60.2% H-5(α) => LUMO(α), 29.0% H-6(β) => L+1(β)
29	31437	318	0.0011	64.9% H-6(β) => L+1(β), 30.4% H-5(α) => LUMO(α)
30	31631	316	0.0170	99.3% HOMO(α) => L+3(α)
32	31745	315	0.0001	61.4% HOMO(α) => L+5(α), 11.0% H-9(β) => LUMO(β)
33	32094	312	0.0012	65.7% H-7(β) => LUMO(β), 18.4% H-3(α) => L+1(α)
34	32143	311	0.0001	26.7% H-7(α) => LUMO(α), 13.5% H-9(β) => LUMO(β), 12.4% HOMO(α) => L+5(α)
37	32887	304	0.0576	41.4% H-3(α) => L+1(α), 30.3% H-7(β) => LUMO(β), 18.9% H-2(β) => L+2(β)
39	33279	301	0.0007	79.2% HOMO(α) => L+8(α), 11.5% HOMO(α) => L+5(α)
40	33718	297	0.0345	97.8% HOMO(α) => L+7(α)
42	34419	291	0.2720	51.6% HOMO(α) => L+9(α), 28.8% H-8(α) => LUMO(α), 16.2% H-7(β) => L+1(β)
45	35832	279	0.1780	67.0% H-2(β) => L+2(β), 31.6% H-3(α) => L+1(α)
49	37534	266	0.0162	46.8% H-10(β) => L+1(β), 29.3% H-11(α) => LUMO(α)
51	38158	262	0.0112	44.5% H-10(β) => LUMO(β), 23.3% H-7(α) => L+1(α), 17.5% H-4(β) => L+2(β)
52	38343	261	0.0016	96.0% HOMO(α) => L+11(α)
54	39146	255	0.0431	93.3% HOMO(α) => L+13(α)
58	40412	247	0.0053	36.4% H-10(β) => LUMO(β), 29.8% H-4(β) => L+2(β), 19.0% H-7(α) => L+1(α)
60	41398	242	0.0355	31.2% H-9(α) => L+1(α), 26.3% H-4(β) => L+2(β), 15.0% H-8(β) => L+2(β), 12.1% H-7(α) => L+1(α)

[a] Only contributions with more than 10% are shown

Table S2. TDDFT predicted energies, oscillator strengths, and contributions for the major excited states for the $[H_4TPeP]^{2+}$ and its redox-active derivatives.

					$[H_4TPeP]^{2+}$
**Excited State**	**Energy (cm^{-1})**	**Wavelength (nm)**	**Osc. Strength**	**Band Assignment**	**Contributions[b]**

1,2	16867	593	0.1300	56.8% HOMO => LUMO, 19.1% HOMO => L+1, 18.0% H-1 => L+1, 56.8% HOMO => L+1, 19.1% HOMO => LUMO, 18.0% H-1 => LUMO
3,4	23515	425	1.5900	70.9% H-1 => LUMO, 23.2% HOMO => L+1, 70.9% H-1 => L+1, 23.2% HOMO => LUMO
5	29389	340	0.0001	80.4% HOMO => L+2
7	30093	332	0.0001	38.1% H-4 => LUMO, 38.1% H-3 => L+1
8,9	30222	331	0.1290	51.1% H-2 => L+1, 27.5% H-2 => LUMO, 17.9% H-5 => LUMO
10	30549	327	0.0030	39.7% H-4 => L+1, 39.7% H-3 => LUMO
12,13	30902	324	0.0262	51.7% H-5 => LUMO, 27.2% H-5 => L+1, 18.0% H-2 => L+1, 51.7% H-5 => L+1, 27.2% H-5 => LUMO, 18.0% H-2 => LUMO
15	32238	310	0.0002	49.7% H-1 => L+2, 14.1% H-7 => L+1, 14.1% H-6 => LUMO
17	34442	290	0.0276	30.8% H-1 => L+2, 30.0% HOMO => L+3, 10.5% H-7 => L+1, 10.5% H-6 => LUMO
18	35093	285	0.0046	22.8% H-7 => LUMO, 22.8% H-6 => L+1, 16.2% H-7 => L+1, 16.2% H-6 => LUMO, 11.2% HOMO => L+2
19,20	37860	264	0.0537	95.7% H-8 => LUMO, 95.7% H-8 => L+1
				$[H_4TPeP]^{\bullet+}$
2,3	13807	724	0.0223	65.9% HOMO(α) => LUMO(α), 13.2% H-5(β) => LUMO(β), 11.2% H-1(α) => L+1(α),65.9% HOMO(α) => L+1(α), 13.2% H-6(β) => LUMO(β), 11.3% H-1(α) => LUMO(α)
4,5	15312	653	0.0016	51.2% H-1(α) => LUMO(α), 14.6% HOMO(β) => L+1(β), 13.7% H-5(β) => LUMO(β),51.3% H-1(α) => L+1(α), 14.6% HOMO(β) => L+2(β), 13.7% H-6(β) => LUMO(β)
7,8	16505	606	0.0013	92.8% H-2(β) => LUMO(β),92.8% H-3(β) => LUMO(β)
9	17113	584	0.0020	97.3% H-4(β) => LUMO(β)
10,11	19163	522	0.2050	48.9% H-5(β) => LUMO(β), 18.9% HOMO(β) => L+1(β), 11.0% HOMO(β) => L+2(β),48.9% H-6(β) => LUMO(β), 18.8% HOMO(β) => L+2(β), 11.0% HOMO(β) => L+1(β)
12	21246	471	0.0014	74.5% H-7(β) => LUMO(β), 10.1% HOMO(α) => L+2(α)
13,14	23538	425	1.3900	52.6% HOMO(β) => L+1(β), 27.1% H-1(α) => LUMO(α),52.6% HOMO(β) => L+2(β), 27.1% H-1(α) => L+1(α)
15	25260	396	0.0005	31.8% H-4(α) => LUMO(α), 26.9% H-3(α) => L+1(α), 12.2% H-3(β) => L+1(β), 10.3% H-2(β) => L+2(β)
17, 18	25350	394	0.0089	55.9% H-2(α) => LUMO(α), 16.7% H-1(β) => L+1(β), 11.8% H-5(α) => LUMO(α),55.9% H-2(α) => L+1(α), 16.7% H-1(β) => L+2(β), 11.7% H-5(α) => L+1(α)
20	26830	373	0.0030	43.8% H-8(β) => LUMO(β), 11.0% H-3(α) => LUMO(α), 10.8% H-4(α) => L+1(α), 10.6% HOMO(α) => L+2(α)
21	26948	371	0.0031	42.5% H-8(β) => LUMO(β), 13.6% H-1(α) => L+2(α)
22	27126	369	0.0013	30.0% HOMO(α) => L+2(α), 13.4% H-3(α) => LUMO(α), 12.4% H-7(β) => LUMO(β), 12.1% H-4(α) => L+1(α)
24	27293	366	0.0029	69.2% H-9(β) => LUMO(β), 13.2% H-5(α) => L+1(α)
25	27295	366	0.0028	68.2% H-10(β) => LUMO(β), 13.9% H-5(α) => LUMO(α)
26	27453	364	0.0014	12.6% H-8(β) => LUMO(β), 12.4% H-4(α) => L+1(α), 12.2% H-1(α) => L+2(α), 12.1% H-3(α) => LUMO(α)
27	27504	364	0.0197	36.2% H-5(α) => LUMO(α), 17.0% H-10(β) => LUMO(β), 12.5% H-9(β) => LUMO(β), 12.0% H-2(α) => LUMO(α), 11.2% H-4(β) => L+1(β)
28	27506	364	0.0196	37.0% H-5(α) => L+1(α), 16.0% H-9(β) => LUMO(β), 12.7% H-10(β) => LUMO(β), 12.1% H-2(α) => L+1(α), 11.4% H-4(β) => L+2(β)
32	28785	347	0.0002	18.0% HOMO(α) => L+2(α), 12.0% H-7(α) => L+1(α), 11.9% H-6(α) => LUMO(α)
33	29330	341	0.0018	87.4% H-13(β) => LUMO(β)
34	29334	341	0.0023	86.6% H-14(β) => LUMO(β)
35,36	29443	340	0.1560	60.8% H-1(β) => L+2(β), 20.4% H-2(α) => L+1(α),60.8% H-1(β) => L+2(β), 20.4% H-2(α) => L+1(α)
37	29717	337	0.0008	15.2% H-3(β) => L+1(β), 13.1% H-2(β) => L+1(β), 12.5% H-3(β) => L+2(β), 12.2% H-2(β) => L+2(β), 11.6% H-1(α) => L+2(α)

39	29876	335	0.0006	22.0% H-1(α) => L+2(α), 17.1% H-3(β) => L+1(β), 17.0% H-2(β) => L+2(β)
40	30062	333	0.0005	97.6% H-15(β) => LUMO(β)
42	30469	328	0.0060	22.0% H-3(β) => L+2(β), 20.5% H-2(β) => L+1(β), 18.4% H-1(α) => L+2(α)
44, 45	30795	325	0.0010	72.7% H-4(β) => L+1(β), 21.7% H-5(α) => LUMO(α),72.4% H-4(β) => L+2(β), 21.6% H-5(α) => L+1(α)
47	32574	307	0.0055	33.1% HOMO(β) => L+3(β), 21.8% H-6(β) => L+2(β), 21.6% H-5(β) => L+1(β)
48,49	32665	306	0.0035	53.8% H-8(α) => LUMO(α), 32.0% H-7(β) => L+1(β),53.8% H-8(α) => L+1(α), 32.0% H-7(β) => L+2(β)
51	33678	297	0.0358	17.2% H-6(β) => L+1(β), 17.2% H-5(β) => L+2(β)
52	34877	287	0.0437	48.1% HOMO(β) => L+3(β), 14.7% HOMO(α) => L+3(α)
53, 54	34938	286	0.0058	93.1% H-17(β) => LUMO(β),93.1% H-18(β) => LUMO(β)
55	36430	275	0.0110	35.7% H-19(β) => LUMO(β), 21.6% H-1(α) => L+3(α), 12.0% HOMO(β) => L+4(β)
56	36430	274	0.0527	49.2% H-7(β) => L+1(β), 32.7% H-8(α) => LUMO(α)
57	36433	274	0.0602	56.9% H-7(β) => L+2(β), 37.3% H-8(α) => L+1(α)
58	37033	270	0.0017	47.2% H-19(β) => LUMO(β), 21.6% HOMO(β) => L+4(β), 11.1% H-1(α) => L+3(α)
59,60	37385	267	0.0077	70.9% H-9(α) => LUMO(α), 20.2% H-9(α) => L+1(α),70.8% H-9(α) => L+1(α), 20.1% H-9(α) => LUMO(α)

[a] Only contributions with more than 10% are shown

Table S3. DFT-predicted molecular orbital compositions for H_2TPeP.[a]

MO	E, eV	Symm	TPeP
93	-9.642	b_2	100
94	-9.602	b_1	100
95	-9.392	*a*	100
96	-9.18	b_3	100
97	-9.122	*a*	100
98	-9.09	b_1	100
99	-9.034	b_3	100
100	-8.887	b_1	100
101	-8.833	b_2	100
102	-8.779	b_2	100
103	-8.048	*a*	100
104	-7.364	b_1	100
105	-7.166	b_3	100
106	-7.05	b_3	100
107	-7.048	b_2	100
108	-7.032	*a*	100
109	-6.893	b_3	100
110	-6.404	b_2	100
111	-6.272	b_1	100
112	-5.405	*a*	100
113	***-4.958***	$\boldsymbol{b_2}$	***100***
114	***-2.75***	$\boldsymbol{b_3}$	***100***
115	-2.704	b_1	100
116	-1.313	*a*	100
117	0.586	b_2	100
118	0.99	b_3	100
119	1.076	b_1	100
120	1.239	*a*	100
121	1.414	b_3	100
122	1.424	*a*	100
123	1.425	b_1	100
124	1.518	b_2	100
125	1.926	*a*	100

126	2.07	b_1	100
127	2.075	*a*	100
128	2.099	b_3	100
129	2.499	b_3	100
130	2.516	b_2	100
131	2.546	b_2	100
132	2.546	*a*	100
133	2.574	b_1	100
134	3.056	b_3	100

a) Frontier orbitals are formatted in bold.

Table S4. DFT-predicted molecular orbital compositions for $[H_2TPeP]^{\bullet+}$. [a]

α-Set				β-Set			
MO	E, eV	Symm	TPeP	MO	E, eV	Symm	TPeP
93	-10.415	b_1	100	92	-10.412	b_2	100
94	-10.394	b_2	100	93	-10.376	b_1	100
95	-10.03	*a*	100	94	-10.328	b_2	100
96	-9.878	b_3	100	95	-10.001	*a*	100
97	-9.795	b_1	100	96	-9.851	b_3	100
98	-9.72	*a*	100	97	-9.77	b_1	100
99	-9.662	b_3	100	98	-9.714	*a*	100
100	-9.593	b_2	100	99	-9.65	b_3	100
101	-9.587	b_1	100	100	-9.568	b_1	100
102	-9.498	b_2	100	101	-9.56	b_2	100
103	-8.954	*a*	100	102	-9.484	b_2	100
104	-8.282	b_1	100	103	-8.736	*a*	100
105	-8.163	b_3	100	104	-8.086	b_1	100
106	-8.137	*a*	100	105	-8.085	b_3	100
107	-8.07	b_3	100	106	-8.061	*a*	100
108	-7.901	b_2	100	107	-7.894	b_3	100
109	-7.847	b_3	100	108	-7.795	b_2	100
110	-7.296	b_2	100	109	-7.67	b_3	100
111	-7.224	b_1	100	110	-7.135	b_2	100
112	-6.232	b_2	100	111	-7	b_1	100
113	***-6.23***	***a***	***100***	***112***	***-6.371***	***a***	***100***
114	***-3.84***	$\boldsymbol{b_1}$	***100***	***113***	***-5.179***	$\boldsymbol{b_2}$	***100***
115	-3.698	b_3	100	114	-3.44	b_3	100
116	-2.282	*a*	100	115	-3.394	b_1	100
117	-0.381	b_2	100	116	-2.011	*A*	100
118	0.212	b_3	100	117	-0.254	b_2	100
119	0.288	b_1	100	118	0.222	b_3	100
120	0.705	*a*	100	119	0.316	b_1	100
121	0.808	*a*	100	120	0.671	*a*	100
122	1.017	b_1	100	121	0.814	*a*	100
123	1.022	b_3	100	122	1.03	b_3	100
124	1.12	b_2	100	123	1.033	b_1	100
125	1.33	*a*	100	124	1.137	b_2	100
126	1.534	*a*	100	125	1.372	*a*	100
127	1.589	b_1	100	126	1.543	*a*	100
128	1.618	b_3	100	127	1.617	b_1	100
129	1.836	*a*	100	128	1.644	b_3	100
130	1.926	b_3	100	129	1.89	*a*	100
131	2.014	b_1	100	130	1.986	b_3	100
132	2.058	b_2	100	131	2.095	b_1	100
133	2.127	b_2	100	132	2.108	b_2	100

a) Frontier orbitals are formatted in bold.

Table S5. DFT-predicted molecular orbital compositions for $[H_2TPeP]^{\bullet -}$. [a]

α-Set				β-Set			
MO	E, eV	Symm	TPeP	MO	E, eV	Symm	TPeP
94	-8.88	b_1	100	93	-8.908	b_2	100
95	-8.776	*a*	100	94	-8.86	b_1	100
96	-8.6	*a*	100	95	-8.764	*a*	100
97	-8.51	b_3	100	96	-8.594	*a*	100
98	-8.465	b_3	100	97	-8.501	b_3	100
99	-8.464	b_1	100	98	-8.451	b_3	100
100	-8.193	b_2	100	99	-8.445	b_1	100
101	-8.16	b_1	100	100	-8.173	b_2	100
102	-8.017	b_2	100	101	-8.147	b_1	100
103	-7.319	*a*	100	102	-8.006	b_2	100
104	-6.664	b_1	100	103	-7.144	*a*	100
105	-6.443	b_3	100	104	-6.48	b_1	100
106	-6.348	b_2	100	105	-6.325	b_3	100
107	-6.13	b_3	100	106	-6.24	b_2	100
108	-6.058	*a*	100	107	-6.064	b_3	100
109	-6.057	b_3	100	108	-6.053	*a*	100
110	-5.448	b_2	100	109	-5.874	b_3	100
111	-5.305	b_1	100	110	-5.294	b_2	100
112	-4.687	*a*	100	111	-5.203	b_1	100
113	-4.262	b_2	100	112	-4.44	*a*	100
114	***-2.577***	$\mathbf{b_3}$	***100***	***113***	***-4.009***	$\mathbf{b_2}$	***100***
115	***-1.823***	$\mathbf{b_1}$	***100***	***114***	***-1.879***	$\mathbf{b_3}$	***100***
116	-0.739	*a*	100	115	-1.746	b_1	100
117	1.362	b_2	100	116	-0.468	*a*	100
118	1.625	*a*	100	117	1.524	b_2	100
119	1.674	b_3	100	118	1.635	*a*	100
120	1.679	b_1	100	119	1.696	b_1	100
121	1.783	b_3	100	120	1.795	b_3	100
122	1.874	b_2	100	121	1.887	b_2	100
123	1.888	b_1	100	122	1.897	b_3	100
124	2.095	*a*	100	123	1.932	b_1	100
125	2.337	*a*	100	124	2.196	*a*	100
126	2.463	b_1	100	125	2.38	*a*	100
127	2.529	b_3	100	126	2.479	b_1	100
128	2.613	*a*	100	127	2.54	b_3	100
129	2.874	b_2	100	128	2.663	*a*	100
130	2.911	b_2	100	129	2.88	b_2	100
131	2.917	b_3	100	130	2.922	b_2	100
132	2.97	b_1	100	131	2.954	b_3	100
133	3.052	*a*	100	132	2.993	b_1	100
134	3.422	b_3	100	133	3.072	*a*	100

a) Frontier orbitals are formatted in bold.

Table S6. DFT-predicted molecular orbital compositions for $[H_4TPeP]^{2+}$. [a]

MO	E, eV	Symm	TPeP
93	-11.198	*a*	100
94,95	-10.956	*e*	100
96	-10.777	*b*	100
97	-10.353	*a*	100
98,99	-10.233	*e*	100
100	-10.08	*b*	100

101	-10.047	*b*	100
102,103	-9.997	*e*	100
104	-9.943	*a*	100
105	-9.49	*a*	100
106,107	-8.827	*e*	100
108	-8.659	*a*	100
109,110	-8.622	*e*	100
111	-8.607	*b*	100
112	-7.271	*b*	100
113	***-6.788***	***b***	***100***
114,115	***-4.622***	***e***	***100***
116	-2.977	*a*	100
117	-1.904	*a*	100
118,119	-0.862	*e*	100
120	-0.448	*a*	100
121	-0.36	*b*	100
122	-0.127	*b*	100
123	0.544	*a*	100
124,125	0.546	*e*	100
126	0.835	*b*	100
127,128	0.973	*e*	100
129	1.063	*a*	100
130	1.136	*b*	100
131,132	1.359	*e*	100
133	1.471	*b*	100
134	1.669	*a*	100

a) Frontier orbitals are formatted in bold.

Table S7. DFT-predicted molecular orbital compositions for [H_4TPeP]$^{\bullet+}$.[a]

α-Set				β-Set			
MO	E, eV	Symm	TPeP	MO	E, eV	Symm	TPeP
93	-11.908	*a*	100	92	-12.222	*a*	100
94,95	-11.668	*a*	100	93	-11.787	*a*	100
96	-11.448	*a*	100	94,95	-11.537	*a*	100
97	-10.953	*a*	100	96	-11.348	*a*	100
98,99	-10.876	*a*	100	97	-10.925	*a*	100
100	-10.769	*a*	100	98,99	-10.849	*a*	100
101	-10.636	*a*	100	100	-10.742	*a*	100
102,103	-10.596	*a*	100	101	-10.627	*a*	100
104	-10.552	*a*	100	102,103	-10.584	*a*	100
105	-10.205	*a*	100	104	-10.538	*a*	100
106,107	-9.638	*a*	100	105	-10.024	*a*	100
108	-9.482	*a*	100	106,107	-9.425	*a*	100
109,110	-9.397	*a*	100	108	-9.386	*a*	100
111	-9.33	*a*	100	109,110	-9.293	*a*	100
112	-8.068	*a*	100	111	-9.22	*a*	100
113	***-7.827***	***a***	***100***	***112***	***-8.175***	***a***	***100***
114,115	***-5.601***	***a***	***100***	***113***	***-6.871***	***a***	***100***
116	-4.052	*a*	100	114,115	-5.253	*a*	100
117	-2.747	*a*	100	116	-3.699	*a*	100
118,119	-1.692	*a*	100	117	-2.714	*a*	100
120	-0.992	*a*	100	118,119	-1.631	*a*	100
121	-0.971	*a*	100	120	-0.946	*a*	100
122	-0.896	*a*	100	121	-0.932	*a*	100
123,124	-0.174	*a*	100	122	-0.901	*a*	100
125	-0.142	*a*	100	123,124	-0.109	*a*	100
126	0.198	*a*	100	125	-0.077	*a*	100

127,128	0.392	*a*	100	126	0.274	*a*	100
129	0.519	*a*	100	127,128	0.428	*a*	100
130	0.534	*a*	100	129	0.557	*a*	100
131	0.794	*a*	100	130	0.569	*a*	100
132,133	0.879	*a*	100	131	0.818	*a*	100

a) Frontier orbitals are formatted in bold.

Table S8. DFT-predicted molecular orbital compositions for ZnTPeP.[a]

MO	E, eV	Symm	Zn	TPeP
107	-9.584	*a*	0	100
108	-9.319	*a*	3.57	96.43
109,110	-9.071	*e*	0.32	99.68
111	-9.066	*b*	0.04	99.96
112,113	-8.984	*e*	0.52	99.48
114	-8.719	*a*	0	100
115	-8.676	*b*	1.92	98.08
116,117	-8.255	*e*	3.71	96.29
118	-7.916	*a*	0	100
119,120	-7.125	*e*	0.09	99.91
121	-6.813	*b*	1.58	98.42
122	-6.393	*a*	0	100
123,124	-6.379	*e*	1.69	98.31
125	-6.065	*b*	16.12	83.88
126	-5.268	*b*	0.02	99.98
127	***-4.921***	***b***	***1.51***	***98.49***
128,129	***-2.585***	***e***	***0.22***	***99.78***
130	-1.209	*a*	0.79	99.21
131	-0.015	*a*	87.27	12.73
132	0.738	*b*	48.94	51.06
133	0.744	*a*	0	100
134,135	1.134	*e*	0.62	99.38
136,137	1.396	*e*	4.16	95.84
138	1.527	*a*	10.02	89.98
139	1.531	*b*	0.03	99.97
140	1.595	*b*	0.56	99.44
141	1.755	*a*	65.47	34.53
142	1.983	*b*	0.01	99.99
143,144	2.092	*e*	2.44	97.56
145	2.272	*a*	8.22	91.78
146	2.553	*a*	0	100
147,148	2.561	*e*	2.34	97.66

a) Frontier orbitals are formatted in bold.

Table S9. DFT-predicted molecular orbital compositions for $[ZnTPeP]^{\bullet+}$.[a]

α-Set					β-Set				
MO	E, eV	Symm	Zn	TPeP	MO	E, eV	Symm	Zn	TPeP
107	-10.34	*b*	0.55	99.45	106	-10.338	*a*	0	100
108	-9.974	*a*	0.59	99.41	107	-10.272	*b*	0.39	99.61
109,110	-9.796	*e*	0.53	99.47	108	-9.944	*a*	0.61	99.39
111	-9.661	*b*	0.03	99.97	109,110	-9.768	*e*	0.54	99.46
112,113	-9.634	*e*	0.67	99.33	111	-9.654	*b*	0.04	99.96
114	-9.474	*b*	1.27	98.73	112,113	-9.62	*e*	0.67	99.33
115	-9.416	*a*	0	100	114	-9.441	*b*	1.24	98.76

116,117	-9.236	*e*	3.06	96.94	115	-9.404	*a*	0	100
118	-8.822	*a*	0	100	116,117	-9.201	*e*	3.16	96.84
119,120	-8.04	*e*	0.02	99.98	118	-8.584	*a*	0	100
121	-7.624	*b*	1.41	98.59	119,120	-7.828	*e*	0.1	99.9
122,123	-7.296	*e*	1.64	98.36	121	-7.521	*b*	1.43	98.57
124	-7.276	*a*	0	100	122	-7.138	*a*	0	100
125	-7.143	*b*	16.59	83.41	123,124	-7.129	*e*	1.44	98.56
126	-6.156	*b*	0.95	99.05	125	-7.091	*b*	16.43	83.57
127	***-6.067***	***b***	***0.02***	***99.98***	***126***	***-6.21***	***b***	***0.02***	***99.98***
128,129	***-3.615***	***e***	***0.18***	***99.82***	***127***	***-5.123***	***b***	***1.41***	***98.59***
130	-2.176	*a*	0.49	99.51	128,129	-3.258	*e*	0.22	99.78
131	-0.449	*a*	81.55	18.45	130	-1.892	*a*	0.56	99.44
132	-0.17	*a*	0.05	99.95	131	-0.423	*a*	82.49	17.51
133	0.109	*b*	50.53	49.47	132	-0.072	*a*	0.03	99.97
134,135	0.386	*e*	0.34	99.66	133	0.16	*b*	49.16	50.84
136	0.843	*b*	0.03	99.97	134,135	0.389	*e*	0.34	99.66
137	0.948	*a*	61.54	38.46	136	0.793	*b*	0.03	99.97
138,139	0.996	*e*	5.24	94.76	137	0.985	*a*	58.17	41.83
140	1.156	*a*	27.06	72.94	138,139	1.009	*e*	5.04	94.96
141	1.222	*b*	0.47	99.53	140	1.175	*a*	30.97	69.03
142	1.568	*b*	0	100	141	1.236	*b*	0.49	99.51
143,144	1.617	*e*	4.18	95.82	142	1.571	*b*	0	100
145	1.656	*a*	4.58	95.42	143,144	1.64	*e*	3.68	96.32
146,147	2.02	*e*	0.15	99.85	145	1.72	*a*	5.16	94.84

a) Frontier orbitals are formatted in bold.

Table S10. DFT-predicted molecular orbital compositions for [ZnTPeP] $^{\bullet-}$.[a]

α-Set					β-Set				
MO	E, eV	Symm	Zn	TPeP	MO	E, eV	Symm	Zn	TPeP
108	-8.671	*a*	19.69	80.31	107	-8.757	*a*	13.46	86.54
109	-8.545	*a*	0.5	99.5	108	-8.658	*a*	19.84	80.16
110	-8.449	b_2	0.13	99.87	109	-8.538	*a*	0.46	99.54
111	-8.405	b_3	0.32	99.68	110	-8.432	b_2	0.13	99.87
113	-8.356	b_3	0.3	99.7	112	-8.352	b_2	0.84	99.16
114	-8.067	b_1	0.35	99.65	113	-8.341	b_3	0.34	99.66
115	-7.924	b_1	2.12	97.88	114	-8.05	b_1	0.3	99.7
116	-7.36	b_2	3.87	96.13	115	-7.911	b_1	2.14	97.86
117	-7.33	b_3	4.27	95.73	116	-7.326	b_2	4.04	95.96
118	-7.185	*a*	0.01	99.99	117	-7.32	b_3	4.28	95.72
119	-6.422	b_2	0.04	99.96	118	-7.006	*a*	0.01	99.99
120	-6.363	b_3	0.18	99.82	119	-6.238	b_2	0.03	99.97
121	-6.067	b_1	1.63	98.37	120	-6.197	b_3	0.35	99.65
122	-5.672	b_3	1.75	98.25	121	-5.943	b_1	1.71	98.29
123	-5.533	b_1	0.15	99.85	122	-5.463	b_3	1.53	98.47
124	-5.453	b_2	1.68	98.32	123	-5.396	b_1	0.22	99.78
125	-5.183	*a*	15.29	84.71	124	-5.343	b_2	1.63	98.37
126	-4.563	*a*	0.03	99.97	125	-5.155	*a*	15.23	84.77
127	-4.253	b_1	1.63	98.37	126	-4.381	*a*	0.02	99.98
128	***-2.418***	$\boldsymbol{b_3}$	***0.24***	***99.76***	***127***	***-3.905***	$\boldsymbol{b_1}$	***1.86***	***98.14***
129	***-1.723***	$\boldsymbol{b_2}$	***0.18***	***99.82***	***128***	***-1.688***	$\boldsymbol{b_3}$	***0.26***	***99.74***
130	-0.646	*a*	1.03	98.97	129	-1.632	b_2	0.18	99.82
131	0.354	*a*	89.35	10.65	130	-0.358	*a*	1.43	98.57
132	1.259	b_1	44.82	55.18	131	0.362	*a*	89.46	10.54
133	1.518	b_1	0.01	99.99	132	1.277	b_1	43.62	56.38
134	1.7	b_2	3.01	96.99	133	1.679	b_1	0.86	99.14
135	1.73	b_3	2.96	97.04	134	1.706	b_2	2.94	97.06
136	1.847	b_3	1.65	98.35	135	1.759	b_3	3.17	96.83

137	1.888	b_2	1.71	98.29	136	1.922	b_2	1.61	98.39
138	1.918	*a*	5.2	94.8	137	1.927	*a*	5.22	94.78
139	1.936	b_1	0.74	99.26	138	1.947	b_1	0.79	99.21
140	2.196	*a*	3.51	96.49	139	2.037	b_3	0.44	99.56
141	2.295	*a*	62.3	37.7	140	2.266	*a*	23.34	76.66
142	2.404	*a*	8.44	91.56	141	2.324	*a*	61	39
143	2.482	b_2	1.47	98.53	142	2.442	*a*	4.43	95.57
144	2.507	b_3	2.7	97.3	143	2.497	b_2	1.35	98.65
145	2.751	*a*	10.42	89.58	144	2.517	b_3	2.68	97.32
146	2.928	*b1*	0.01	99.99	145	2.798	*a*	10.18	89.82
147	2.955	b_1	0.27	99.73	146	2.936	b_1	0.02	99.98
148	2.958	b_2	2.13	97.87	147	2.967	b_1	0.28	99.72
149	2.966	b_3	0.78	99.22	148	2.98	b_2	2.53	97.47

a) Frontier orbitals are formatted in bold.

Table S11. TDDFT predicted energies, oscillator strengths, and contributions for the major excited states for the neutral ZnTPeP complex and its redox-active derivatives.

Excited State	Energy (cm^{-1})	Wavelength (nm)	Osc. Strength	Band Assignment	Contributions[b]
					**ZnTPeP**
1,2	17992	556	0.0575	Q	65.0% HOMO → L+1, 27.2% H-1 → LUMO [65.0% HOMO → LUMO, 27.2% H-1 → L+1]
3,4	23538	425	1.5700	Soret	51.5% H-1 → L+1, 21.6% HOMO → LUMO, 18.9% H-1 → LUMO [51.5% H-1 → LUMO, 21.6% HOMO → L+1, 18.9% H-1 → L+1]
15,16	32293	310	0.1560		96.5% H-6 → L+1 [96.5% H-6 → LUMO]
					**$[ZnTPeP]^{+\cdot}$**
3,4	12622	792	0.0011	"1*e*" → a_{2u}	89.6% H-2(β) → LUMO(β) [89.6% H-3(β) → LUMO(β)]
6,7	15460	647	0.0030	a_{1u} → e_g	42.9% HOMO(α) → L+1(α), 27.7% HOMO(α) → LUMO(α), 11.0% HOMO(β) → L+2(β) [42.9% HOMO(α) → LUMO(α), 27.7% HOMO(α) → L+1(α), 11.0% HOMO(β) → L+1(β)]
9,10	16511	606	0.0068	"2*e*" → a_{2u} + a_{1u} → e_g	42.1% H-6(β) → LUMO(β), 35.3% H-1(α) → LUMO(α) [42.1% H-7(β) → LUMO(β), 35.3% H-1(α) → L+1(α)]
11,12	20200	495	0.0962	a_{2u}/a_{1u} → e_g + "2*e*" → a_{2u}	36.8% H-7(β) → LUMO(β), 26.5% HOMO(β) → L+1(β), 20.7% H-1(α) → L+1(α) [36.8% H-6(β) → LUMO(β), 26.5% HOMO(β) → L+2(β), 20.7% H-1(α) → LUMO(α)]

14,15	23798	420	1.3200	Soret	34.9% HOMO(β) → L+2(β), 11.0% HOMO(α) → LUMO(α), 10.7% HOMO(α) → L+1(α) [34.9% HOMO(β) → L+1(β), 11.0% HOMO(α) → L+1(α), 10.7% HOMO(α) → LUMO(α)]
20,21	24694	405	0.1780		28.9% H-2(α) → LUMO(α), 23.7% H-3(α) → L+1(α), 11.6% H-3(α) → LUMO(α), 10.8% H-4(β) → L+2(β) [28.9% H-2(α) → L+1(α), 23.7% H-3(α) → LUMO(α), 11.6% H-3(α) → L+1(α), 10.8% H-4(β) → L+1(β)]
44,45	31777	315	0.1360		71.4% H-5(β) → L+1(β), 20.5% H-6(α) → LUMO(α) [71.4% H-5(β) → L+2(β), 20.5% H-6(α) → L+1(α)]
**[ZnTPeP]$^{-\cdot}$**					
2	10498	953	0.0832	"e_{gx}" → "e_{gy}" + a_{2u} → "e_{gy}"	63.7% HOMO(α) → L+1(α), 30.4% HOMO(β) → L+1(β)
3	14103	709	0.0539	a_{2u} → "e_{gy}"+ "e_{gx}" → "e_{gy}"	36.0% HOMO(β) → L+1(β), 29.2% HOMO(α) → L+1(α), 27.2% H-1(α) → LUMO(α)
4	14962	668	0.1310	Q	90.1% HOMO(β) → LUMO(β)
5	17627	567	0.0121	a_{1u} → "e_{gy}"	50.5% H-1(β) → L+1(β), 43.8% H-2(α) → LUMO(α)
8	21823	458	1.4000	Soret	38.2% H-1(β) → LUMO(β), 26.4% HOMO(β) → L+1(β), 26.4% H-1(α) → LUMO(α)
9	22548	443	0.7310	Soret	53.8% H-2(α) → LUMO(α), 35.7% H-1(β) → L+1(β)
16	25465	393	0.0375		43.8% H-4(β) → L+1(β), 37.0% H-5(α) → LUMO(α)
18	25884	386	0.0220		90.5% H-4(β) → LUMO(β)
19	26598	376	0.0238		96.6% HOMO(α) → L+3(α)
26	28942	346	0.6530		82.1% HOMO(α) → L+4(α)
37	32641	306	0.0257		98.5% HOMO(α) → L+9(α)

38	32938	304	0.0040	98.8% HOMO(α) → L+10(α)
39	33008	303	0.1370	48.8% H-7(α) → LUMO(α), 48.3% H-6(β) → L+1(β)
42	34086	293	0.1890	90.9% HOMO(α) → L+11(α)

[a)] Only contributions with more than 10% are shown

Excited State	Energy (cm^{-1})	Wavelength (nm)	Osc. Strength	Band Assignment	Contributions[a]
			H_2TPeP		
1	16799	595	0.0875	Q_x	77.5% HOMO → LUMO, 22.1% H-1 → L+1
2	17954	557	0.0799	Q_y	72.5% HOMO → L+1, 27.3% H-1 → LUMO
3	23326	429	1.3600	Soret	75.2% H-1 → L+1, 20.7% HOMO → LUMO, 4.94% H-3 → LUMO
4	23557	425	1.6300	Soret	72.5% H-1 → LUMO, 28.2% HOMO → L+1
7	27816	360	0.4010		94.2% H-3 → LUMO, 2.82% H-1 → L+1, 2.65% HOMO → LUMO
17	33069	302	0.0954		99.0% H-6 → LUMO
18	331134	302	0.1060		98.8% H-6 → L+1
			$[H_4TPeP]^{2+}$		
1,2	16867	593	0.1300	Q	56.8% HOMO → LUMO, 19.1% HOMO → L+1, 18.0% H-1 → L+1 [56.8% HOMO → L+1, 19.1% HOMO → LUMO, 18.0% H-1 → LUMO]

3,4	23515	425	1.5900	Soret	70.9% H-1 → LUMO, 23.2% HOMO → L+1 [70.9% H-1 → L+1, 23.2% HOMO → LUMO
8,9	30222	331	0.1290		51.1% H-2 → L+1, 27.5% H-2 → LUMO, 17.9% H-5 → LUMO [51.1% H-2 → L+1, 27.5% H-2 → LUMO, 17.9% H-5 → LUMO]
12,13	30902	324	0.0262		51.7% H-5 → LUMO, 27.2% H-5 → L+1, 18.0% H-2 → L+1 [51.7% H-5 → L+1, 27.2% H-5 → LUMO, 18.0% H-2 → LUMO]
17	34442	290	0.0276		30.8% H-1 → L+2, 30.0% HOMO → L+3, 10.5% H-7 → L+1, 10.5% H-6 → LUMO
19,20	37860	264	0.0537		95.7% H-8 → LUMO [95.7% H-8 → L+1]
			$[H_2TPeP]^{-\cdot}$		
2	10503	952	0.0684	a_{2u} → "e_{gy}"+ "e_{gx}" → "e_{gy}"	43.7% HOMO(α) → L+1(α), 43.3% HOMO(β) → L+1(β), 13.9% H-1(α) → LUMO(α)
3	13437	744	0.0468	"e_{gx}" → "e_{gy}" + a_{2u} → "e_{gy}"	45.9% HOMO(α) → L+1(α), 22.7% H-1(α) → LUMO(α), 19.0% HOMO(β) → L+1(β), 12.2% H-1(β) → LUMO(β)
4	14453	692	0.1090	Q	93.0% HOMO(β) → LUMO(β)
6	17631	567	0.0180		61.2% H-1(β) → L+1(β), 35.6% H-2(α) → LUMO(α)
7	21643	462	1.5600	Soret	33.4% HOMO(β) → L+1(β), 28.9% H-1(β) → LUMO(β), 28.8% H-1(α) → LUMO(α)
8	22410	446	0.5160	Soret	53.5% H-2(α) → LUMO(α), 21.9% H-1(β) → L+1(β), 15.8% H-3(β) → LUMO(β)
12	23529	425	0.3560		76.5% H-3(β) → LUMO(β)
17	27204	368	0.0810		62.6% H-4(α) → LUMO(α), 36.3% H-3(β) → L+1(β)
20	29161	343	0.6610		86.6% HOMO(α) → L+2(α)

21	29277	342	0.0225	42.8% H-7(β) → L+1(β), 39.9% H-8(α) → LUMO(α)
30	31631	316	0.0170	99.3% HOMO(α) → L+3(α)
37	32887	304	0.0576	41.4% H-3(α) → L+1(α), 30.3% H-7(β) → LUMO(β), 18.9% H-2(β) → L+2(β)
40	33718	297	0.0345	97.8% HOMO(α) → L+7(α)
42	34419	291	0.2720	51.6% HOMO(α) → L+9(α), 28.8% H-8(α) → LUMO(α), 16.2% H-7(β) → L+1(β)
45	35832	279	0.1780	67.0% H-2(β) → L+2(β), 31.6% H-3(α) → L+1(α)

[a] Only contributions with more than 10% are shown

Table S12. Crystal data and structure refinement for Cu(TpeP).

Identification code	Cu(TpeP)	
Empirical formula	$C_{46}H_{64}CuN_4$	
Formula weight	736.55	
Temperature	100(2) K	
Wavelength	0.71073 Å	
Crystal system	Triclinic	
Space group	P-1	
Unit cell dimensions	a = 4.9302(15) Å	α= 100.345(9)°.
	b = 14.258(4) Å	β= 95.766(9)°.
	c = 14.362(4) Å	γ = 92.509(9)°.
Volume	986.1(5) $Å^3$	
Z	1	
Density (calculated)	1.240 Mg/m^3	
Absorption coefficient	0.591 mm^{-1}	
F(000)	397	
Crystal size	0.372 x 0.146 x 0.104 mm^3	
Theta range for data collection	1.854 to 26.499°.	
Index ranges	-6<=h<=6, -17<=k<=17, -17<=l<=17	
Reflections collected	30990	
Independent reflections	4045 [R(int) = 0.1195]	
Completeness to theta = 25.242°	99.9 %	
Absorption correction	SADABS	
Refinement method	Full-matrix least-squares on F^2	
Data / restraints / parameters	4045 / 0 / 234	
Goodness-of-fit on F^2	1.030	
Final R indices [I>2sigma(I)]	R1 = 0.0899, wR2 = 0.2231	
R indices (all data)	R1 = 0.1245, wR2 = 0.2660	
Largest diff. peak and hole	1.700 and -2.062 e.$Å^{-3}$	
